# Clinical characteristics of children and young people admitted to hospital with covid-19 in United Kingdom: prospective multicentre observational cohort study

**DOI:** 10.1136/bmj.m3249

**Published:** 2020-08-27

**Authors:** Olivia V Swann, Karl A Holden, Lance Turtle, Louisa Pollock, Cameron J Fairfield, Thomas M Drake, Sohan Seth, Conor Egan, Hayley E Hardwick, Sophie Halpin, Michelle Girvan, Chloe Donohue, Mark Pritchard, Latifa B Patel, Shamez Ladhani, Louise Sigfrid, Ian P Sinha, Piero L Olliaro, Jonathan S Nguyen-Van-Tam, Peter W Horby, Laura Merson, Gail Carson, Jake Dunning, Peter J M Openshaw, J Kenneth Baillie, Ewen M Harrison, Annemarie B Docherty, Malcolm G Semple

**Affiliations:** 1 Department of Child Life and Health, University of Edinburgh, Edinburgh, UK; 2 Royal Hospital for Sick Children, Paediatric Infectious Diseases, Edinburgh, UK; 3 Women’s and Children’s Health, Institute of Translational Medicine, Faculty of Health and Life Sciences, University of Liverpool, Liverpool, UK; 4 Respiratory Medicine, Alder Hey Children’s NHS Foundation Trust, Liverpool L12 2AP, UK; 5 Institute of Infection, Veterinary and Ecological Sciences, Faculty of Health and Life Sciences, University of Liverpool, Liverpool, UK; 6 Infectious diseases Unit, Royal Liverpool University Hospital, Liverpool, UK; 7 Paediatric Infectious Diseases, Royal Hospital for Children, Glasgow, UK; 8 Centre for Medical Informatics, Usher Institute, University of Edinburgh, Edinburgh, UK; 9 Institute for Adaptive and Neural Computation, School of Informatics, University of Edinburgh, UK; 10 Liverpool Clinical Trials Centre, University of Liverpool, Liverpool, UK; 11 Centre for Tropical Medicine and Global Health, Nuffield Department of Medicine, University of Oxford, Oxford, UK; 12 Respiratory Medicine, Alder Hey Children’s Hospital, Liverpool, UK; 13 Immunisation and Countermeasures Division, Public Health England, Colindale, UK; 14 Paediatric Infectious Disease, St George’s Hospital, London, UK; 15 ISARIC Global Support Centre, Centre for Tropical Medicine and Global Health, Nuffield Department of Medicine, University of Oxford, Oxford, UK; 16 Division of Epidemiology and Public Health, University of Nottingham School of Medicine, Nottingham, UK; 17 United Kingdom Department of Health and Social Care, London, UK; 18 National Infection Service, Public Health England, **[A: Where?]**; 19 National Heart and Lung Institute, Imperial College London, London, UK; 20 Roslin Institute, University of Edinburgh, Edinburgh, UK; 21 Intensive Care Unit, Royal Infirmary Edinburgh, Edinburgh, UK

## Abstract

**Objective:**

To characterise the clinical features of children and young people admitted to hospital with laboratory confirmed severe acute respiratory syndrome coronavirus 2 (SARS-CoV-2) infection in the UK and explore factors associated with admission to critical care, mortality, and development of multisystem inflammatory syndrome in children and adolescents temporarily related to coronavirus disease 2019 (covid-19) (MIS-C).

**Design:**

Prospective observational cohort study with rapid data gathering and near real time analysis.

**Setting:**

260 hospitals in England, Wales, and Scotland between 17 January and 3 July 2020, with a minimum follow-up time of two weeks (to 17 July 2020).

**Participants:**

651 children and young people aged less than 19 years admitted to 138 hospitals and enrolled into the International Severe Acute Respiratory and emergency Infections Consortium (ISARIC) WHO Clinical Characterisation Protocol UK study with laboratory confirmed SARS-CoV-2.

**Main outcome measures:**

Admission to critical care (high dependency or intensive care), in-hospital mortality, or meeting the WHO preliminary case definition for MIS-C.

**Results:**

Median age was 4.6 (interquartile range 0.3-13.7) years, 35% (225/651) were under 12 months old, and 56% (367/650) were male. 57% (330/576) were white, 12% (67/576) South Asian, and 10% (56/576) black. 42% (276/651) had at least one recorded comorbidity. A systemic mucocutaneous-enteric cluster of symptoms was identified, which encompassed the symptoms for the WHO MIS-C criteria. 18% (116/632) of children were admitted to critical care. On multivariable analysis, this was associated with age under 1 month (odds ratio 3.21, 95% confidence interval 1.36 to 7.66; P=0.008), age 10-14 years (3.23, 1.55 to 6.99; P=0.002), and black ethnicity (2.82, 1.41 to 5.57; P=0.003). Six (1%) of 627 patients died in hospital, all of whom had profound comorbidity. 11% (52/456) met the WHO MIS-C criteria, with the first patient developing symptoms in mid-March. Children meeting MIS-C criteria were older (median age 10.7 (8.3-14.1) *v* 1.6 (0.2-12.9) years; P<0.001) and more likely to be of non-white ethnicity (64% (29/45) *v* 42% (148/355); P=0.004). Children with MIS-C were five times more likely to be admitted to critical care (73% (38/52) *v* 15% (62/404); P<0.001). In addition to the WHO criteria, children with MIS-C were more likely to present with fatigue (51% (24/47) *v* 28% (86/302); P=0.004), headache (34% (16/47) *v* 10% (26/263); P<0.001), myalgia (34% (15/44) *v* 8% (21/270); P<0.001), sore throat (30% (14/47) *v* (12% (34/284); P=0.003), and lymphadenopathy (20% (9/46) *v* 3% (10/318); P<0.001) and to have a platelet count of less than 150 × 10^9^/L (32% (16/50) *v* 11% (38/348); P<0.001) than children who did not have MIS-C. No deaths occurred in the MIS-C group.

**Conclusions:**

Children and young people have less severe acute covid-19 than adults. A systemic mucocutaneous-enteric symptom cluster was also identified in acute cases that shares features with MIS-C. This study provides additional evidence for refining the WHO MIS-C preliminary case definition. Children meeting the MIS-C criteria have different demographic and clinical features depending on whether they have acute SARS-CoV-2 infection (polymerase chain reaction positive) or are post-acute (antibody positive).

**Study registration:**

ISRCTN66726260.

## Introduction

Children and young people comprise only 1-2% of cases of coronavirus disease 2019 (covid-19) worldwide.^1^ ^2^ ^3^ In contrast to other respiratory viruses, children seem to have a lower risk of infection than adults,^4^ and the vast majority of reported infections in children are mild or asymptomatic, with few recorded childhood fatalities attributed to covid-19.^2^ ^5^ ^6^ ^7^ Initial reports from China showed that only 0.6% of children with covid-19 were critically ill.^5^

A severe disease phenotype has emerged in children that seems to be temporally associated with severe acute respiratory syndrome coronavirus 2 (SARS-CoV-2) infection.^8^ ^9^ The condition was first described in May 2020 in a cluster of children admitted to critical care in south London (UK), with evidence of a multisystem hyperinflammatory state with features similar to Kawasaki disease and toxic shock syndrome.^8^ These children needed inotropic support for refractory circulatory shock and mechanical ventilation for cardiovascular stabilisation rather than respiratory failure. Similar cohorts have been reported in Italy and France.^10^ ^11^ The European Centre for Disease Prevention and Control estimated on 15 May 2020 that around 230 children had presented with this new syndrome in EU/EEA countries, with two fatalities.^3^ The World Health Organization and the Royal College of Paediatrics and Child Health (RCPCH) have proposed preliminary case definitions.^9^ ^12^ WHO uses the term multisystem inflammatory syndrome in children and adolescents temporarily related to covid-19 (MIS-C), and the RCPCH describes this illness as paediatric inflammatory multisystem syndrome temporally associated with SARS-CoV-2 (PIMS-TS).

We aimed to characterise the features of children and young people (aged <19 years) admitted to hospital in the UK with laboratory confirmed SARS-CoV-2 infection from the International Severe Acute Respiratory and emerging Infection Consortium (ISARIC) WHO Clinical Characterisation Protocol UK (CCP-UK) cohort. As our study enrolled patients prospectively from the beginning of the pandemic, we also had a unique opportunity to examine the emergence, timing, risk factors, clinical presentation, progression, course, and outcomes of children and young people meeting the WHO preliminary case definition for MIS-C.^9^

## Methods

### Study design and setting

The ISARIC WHO CCP-UK is an ongoing prospective cohort study across acute care hospitals in England, Wales, and Scotland.^13^ This standing protocol for studying disease caused by pathogens of public health interest was activated on 17 January 2020. The protocol, associated documents, and details of the Independent Data and Material Access Committee (IDAMAC) are available at https://isaric4c.net. We used STROBE guidelines when reporting.

### Participants

Patients of any age admitted to hospital with proven or high likelihood of SARS-CoV-2 infection were enrolled into the ISARIC WHO CCP-UK cohort as previously described.^13^ We present the data from children and young people aged less than 19 years on the date of hospital admission, enrolled into the study up to and including 3 July 2020, who had at least two weeks of outcome data available. For this report, we included only those children and young people who had documented laboratory evidence of SARS-CoV-2 infection (by polymerase chain reaction or serology). Patients were admitted to hospital or critical care at the discretion of the clinical team caring for them, and we set no criteria for these. We also included patients who were already admitted for other clinical reasons and subsequently tested positive for SARS-CoV-2 while an inpatient.

### Data collection

Demographic and baseline data (including comorbidities and regular medications taken) alongside data on symptoms, clinical signs (including vital signs) during admission, laboratory and pathology investigations, treatments received while admitted, and outcome were collected onto case report forms (see supplementary methods). Data on illness progression and severity, including location within the hospital (ward versus critical care), were collected on day 1 (admission/diagnosis), day 3, day 6, day 9, admission to critical care, and discharge/death. Data were collected from healthcare records onto the case report forms through a secure online database, REDCap (Research Electronic Data Capture, Vanderbilt University, hosted by the University of Oxford). Collection of this routine anonymised demographic and clinical data from medical records did not require consent in England and Wales. In Scotland, a waiver for consent was obtained from the Public Benefit and Privacy Panel.

### Variables

The case report form was agnostic to patients’ age, so existing comorbidity variables were not tailored to the paediatric population (see supplementary methods for case report form and recoding of paediatric variables). Ethnicity was self-reported and transcribed from the healthcare record. The Paediatric Early Warning Score (PEWS) was used as a measure of disease severity at admission.^14^

### Criteria for diagnosis of MIS-C

We used the WHO preliminary case definition for MIS-C as a framework for identifying children with the syndrome within this dataset (box 1),^9^ with adaptations defining thresholds to allow case identification in the ISARIC WHO CCP-UK cohort.

Box 1 **WHO preliminary case definition for MIS-C, with adaptations (italics) defining thresholds to allow case identification in ISARIC WHO CCP-UK cohort**

1. Fever for 3 days or more (*of any duration, self-reported before presentation*)
2. Plus two of the following:
  - Rash or bilateral non-purulent conjunctivitis or muco-cutaneous inflammatory signs (*self-reported rash/conjunctivitis*)
  - Hypotension or shock (*age <2 years, systolic blood pressure <60 mm Hg; ≥2 and <5 years, <70 mm Hg; ≥5 and <12 years, <80 mm Hg; ≥12 years, <90 mm Hg at any point in admission* 14)
  - Features of myocardial dysfunction, pericarditis, valvulitis, or coronary abnormalities (*diagnosis of endocarditis or myocarditis or documented pericardial effusion, coronary artery aneurysm, cardiomegaly, or cardiac dysfunction on echocardiography*)
  - Evidence of coagulopathy (*international normalised ratio >1.2 (any age),* *premature neonate with prothrombin time >16 s* 15 *or term infant/older child with prothrombin time >14 s* 16 17 18 (see supplementary methods))
  - Acute gastrointestinal problems (*self-reported diarrhoea, vomiting, or abdominal pain*)
3. Plus elevated markers of inflammation (*C reactive protein ≥60 mg/L or ferritin ≥200 mg/L at any point during admission; cut-offs chosen after expert discussion)*
4. Plus no other obvious microbial cause of inflammation (*no significant positive growth on blood culture/cerebrospinal fluid culture during admission*)

### Duplicates

For cases in which a child appeared twice in the dataset (that is, by readmission or transfer between two recruiting centres), we retained only one admission in the data (detailed in supplementary methods).

### Outcomes

The primary outcomes of this study were admission to critical care (high dependency unit or paediatric intensive care unit), development of MIS-C, and in-hospital mortality. Paediatric intensive care units are dedicated care settings providing the highest level of critical care for children and young people, who usually need invasive ventilation or support for two or more organs with a higher nurse to patient ratio. Paediatric intensive care units are usually located in regional tertiary centres or specialised hospitals. Paediatric high dependency units are for patients needing close monitoring and therapies for organ support without invasive ventilation or intensive care and are provided at tertiary hospitals and a limited number of district general hospitals.^19^ Admission to critical care is governed by the degree of physiological instability rather than diagnosis. We also examined need for respiratory and cardiovascular support. A minimum two week follow-up was ensured for all included patients.

### Bias and missing data

Specialist children’s hospitals (tertiary care) with paediatric specific research teams and paediatric intensive care units may be over-represented. Capacity to enrol was also limited by staff resources at times of high covid-19 activity, and we were unable to comment on patients who were not recruited.

The pandemic disrupted routine care and usual research activities, limiting data collection and verification, particularly during the peak of outbreak activity. We did not impute missing data for this descriptive analysis. To reduce the effect of missing data on outcome analyses, we restricted these analyses to patients who had been admitted for at least two weeks before data extraction. Complete data were not available for all variables, so denominators differ between analyses.

### Statistical analysis

Continuous variables are shown as mean (standard deviation) or as median (interquartile range) if non-normally distributed. Categorical variables are presented as a frequency (percentage) unless otherwise stated. For univariable comparisons, we used Welch’s *t*, analysis of variance, Mann-Whitney U, or Kruskal-Wallis tests, according to data distribution. We compared categorical data by using χ^2^ tests and considered a P value below 0.05 to be statistically significant; all tests were two sided. We made no adjustment for multiple comparisons. Parsimonious criterion based model building used the following principles: relevant explanatory variables were identified from previous studies; interactions were checked at first order level; final model selection was informed by the Akaike information criterion and C statistic, with appropriate assumptions checked including the distribution of residuals. Analysis of symptom co-occurrence used the Jaccard similarity coefficient and was presented as a hierarchically ordered heatmap (supplementary methods). We used R (R Core Team version 3.6.3, Vienna, Austria) for statistical analyses, with packages including tidyverse, finalfit lubridate, ggplot2, gplot, mclust, dendextend, and UpSetR.

### Patient and public involvement

This was an urgent public health research study in response to a public health emergency of international concern. Patients and the public were not involved in the design, conduct, or reporting of this rapid response research.

## Results

Between 17 January and 3 July 2020, 69 516 patients of all ages (range 0-106 years) were enrolled at 260 hospitals across England, Scotland, and Wales. Of these, 651 were patients under 19 years old with laboratory confirmed SARS-CoV-2 (651/69 516 (0.9%) of the total cohort (table 1; supplementary figures A and B). These patients were enrolled across 138 sites, of which 20 had a paediatric intensive care unit. We recruited 55 patients as hospital inpatients; they had been admitted for more than five days before symptom onset, indicating likely hospital acquired infection.

**Table 1:** Demographics across cohort of patients under 19 years with laboratory confirmation of SARS-CoV-2. Values are numbers (percentages) unless stated otherwise

Variable	Patients (n=651)
Median (interquartile range) age, years	4.6 (0.3-13.7)
Age group:	
<1 month	53 (8.1)
1 month to <1 year	172 (26.4)
1-4 years	108 (16.6)
5-9 years	92 (14.1)
10-14 years	94 (14.4)
15-19 years	132 (20.3)
Sex at birth:	
Male	367 (56.4)
Female	283 (43.5)
Missing	1 (0.2)
Ethnicity:	
White	330 (50.7)
Black	56 (8.6)
South Asian	67 (10.3)
Other	123 (18.9)
Missing	75 (11.5)
Admitted >5 days before symptom onset:	
No	538 (82.6)
Yes	55 (8.4)
Missing	58 (8.9)
Any comorbidity:	
No/unknown	375 (57.6)
Yes	276 (42.4)
Neurological comorbidity:	
No	549 (84.3)
Yes	65 (10.0)
Missing	37 (5.7)
Haematological, oncological, immunological comorbidity:	
No	567 (87.1)
Yes	48 (7.4)
Missing	36 (5.5)
Asthma:	
No	570 (87.6)
Yes	45 (6.9)
Missing	36 (5.5)
Prematurity*:	
No	155 (23.8)
Yes	46 (7.1)
Missing	450 (69.1)
Immunosuppressant use before presentation†:	
No	546 (83.9)
Yes	53 (8.1)
Missing	52 (8.0)

* Defined as birth before completion of 37 weeks’ gestation.

† Includes oral but not inhaled corticosteroids.

### Age, sex, and ethnicity

The median age of the children was 4.6 (interquartile range 0.3-13.7) years, 35% (225/651) were under 12 months old, and 56% (367/650) were male (table 1). Ethnicity was recorded in 88% (576/651) of cases: 57% (330/576) were white, 12% (67/576) were South Asian, and 10% (56/576) were of black ethnicity. At least one comorbidity was reported in 42% (276/651) of cases.

### Symptoms

**Fig 1:**
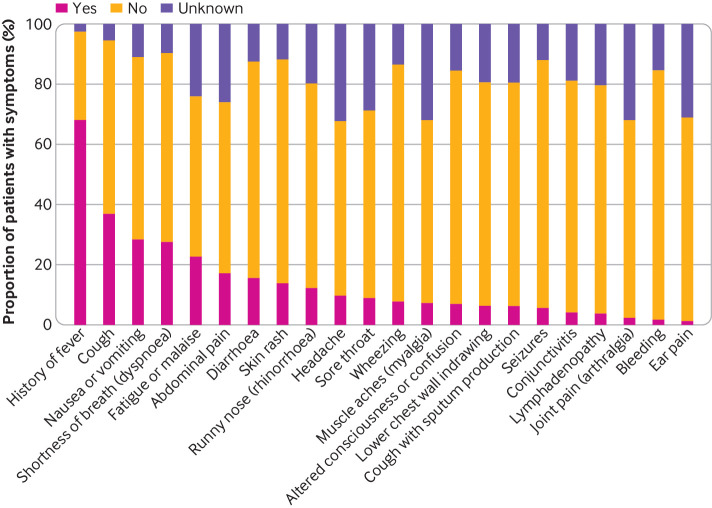
Proportion of patients presenting with each symptom

**Fig 2:**
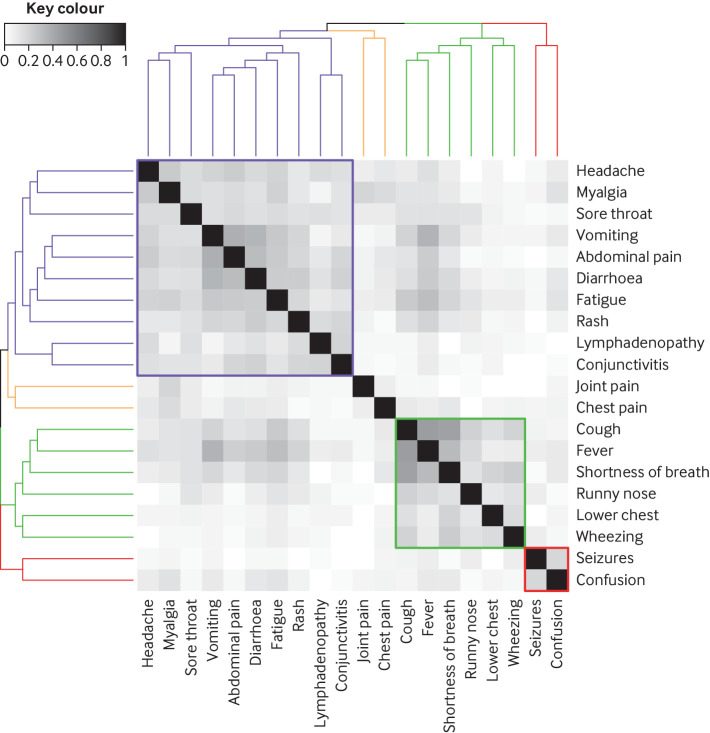
Heatmap with dendrogram describing clusters (coloured) of co-occurring symptoms calculated using hierarchical clustering with Jaccard distance as metric and complete linkage. Heatmap shows pairwise Jaccard indices among 20 symptoms. Jaccard index is measure of similarity that calculates ratio of number of times two symptoms appear together in data and number of times either of them appears in data. Index varies between 0 and 1, with 0 implying that two symptoms never appear together (no co-occurrence) and 1 implying that two symptoms appear only together (co-occurrence only). Dendrogram describing clusters of symptoms in heatmap was calculated using hierarchical clustering with Jaccard distance as metric and complete linkage, where Jaccard distance is calculated by subtracting Jaccard index from 1. Lower chest=lower chest wall indrawing

The most common presenting symptoms were fever (70%; 431/617), cough (39%; 233/599), nausea/vomiting (32%; 179/564), and shortness of breath (30%; 173/570) (fig 1). Fever and rhinorrhoea were less common with increasing age; however, nausea and vomiting, abdominal pain, headache, and sore throat showed an increasing trend with age (supplementary figure D). A heatmap and dendrogram of presenting symptoms showed three distinct clusters of clinical phenotypes (fig 2). These comprised most commonly a discrete respiratory illness (green cluster) of cough, fever, shortness of breath, runny nose, lower chest wall indrawing, and wheeze, with clustering of both upper and lower respiratory symptoms together. Next was a cluster representing a systemic mucocutaneous-enteric illness (purple cluster) of headache, myalgia, sore throat, vomiting, abdominal pain, diarrhoea, fatigue, rash, lymphadenopathy, and conjunctivitis. Finally, we observed a rarer neurological cluster of seizures and confusion (red cluster). The systemic mucocutaneous-enteric cluster includes the symptoms specified in the WHO preliminary case definition for MIS-C (“muco-cutaneous inflammation” and “acute gastrointestinal problems”), in addition to sore throat, myalgia, headache, and fatigue. The two main clusters “respiratory” and “systemic mucocutaneous-enteric” were not entirely dichotomous. Minor overlap occurred between a sub-cluster of “fever, cough, and shortness of breath” and a sub-cluster of “vomiting, abdominal pain, diarrhoea, fatigue, and rash” but very little overlap with “runny nose, wheeze, and lower chest wall indrawing.”

### Comorbidities

The most common comorbidities were neurological (11%; 65/614), haematological/oncological/immunological (combined category as described in supplementary methods: 8%; 48/615), and asthma (7%; 45/615). Data on prematurity (defined as birth before completion of 37 weeks’ gestation) were as usual routinely collected only for children aged under 1 year, and 23% (46/201) were premature (table 1, supplementary table A, and supplementary figure C).

The median PEWS at presentation was 3 (interquartile range 1.0-5.0), and median blood test results at presentation were mostly within normal ranges (supplementary tables B and C). Antibiotics were given to 69% (415/601) of children, and 6% (38/591) received antiviral drugs (30 received acyclovir, 7 received remdesivir, and 3 received chloroquine/hydroxychloroquine, some in combination) (table 2).

**Table 2:** Treatments received and outcomes. Values are numbers (percentages)

	Patients (n=651)
**Treatment**	
Antibiotic:	
No	186 (28.6)
Yes	415 (63.7)
Missing	50 (7.7)
Intravenous steroid:	
No	503 (77.3)
Yes	47 (7.2)
Missing	101 (15.5)
Oral steroid:	
No	503 (77.3)
Yes	30 (4.6)
Missing	118 (18.1)
Antiviral drug:	
No	553 (84.9)
Yes	38 (5.8)
Missing	60 (9.2)
Supplementary oxygen:	
No	455 (69.9)
Yes	172 (26.4)
Missing	24 (3.7)
High flow nasal cannula oxygen:	
No	536 (82.3)
Yes	76 (11.7)
Missing	39 (6.0)
Non-invasive ventilation:	
No	562 (86.3)
Yes	57 (8.8)
Missing	32 (4.9)
Invasive ventilation:	
No	562 (86.3)
Yes	58 (8.9)
Missing	31 (4.8)
Inotropic support:	
No	550 (84.5)
Yes	47 (7.2)
Missing	54 (8.3)
Nursed prone:	
No	574 (88.2)
Yes	15 (2.3)
Missing	62 (9.5)
Nitric oxide:	
No	583 (89.6)
Yes	7 (1.1)
Missing	61 (9.4)
ECMO:	
No	613 (94.2)
Yes	2 (0.3)
Missing	36 (5.5)
Dialysis/haemofiltration:	
No	593 (91.1)
Yes	5 (0.8)
Missing	53 (8.1)
ICU/HDU admission:	
No	516 (79.3)
Yes	116 (17.8)
Missing	19 (2.9)
**Outcome**	
Died	6 (0.9)
Discharged alive	557 (85.6)
Ongoing care	62 (9.5)
Palliative discharge	2 (0.3)
Missing	24 (3.7)

ECMO=extracorporeal membrane oxygenation; HDU=high dependency unit; ICU=intensive care unit.

### Children needing critical care

Eighteen per cent (116/632) of children were admitted to critical care (intensive care unit or high dependency unit level care); 8% (47/597) received inotropic support, 9% (57/619) received non-invasive ventilation, and 9% (58/620) received invasive mechanical ventilation (table 2, table 3, and supplementary figure E). Black ethnicity was significantly associated with admission to critical care on multivariable analysis (odds ratio 2.82, 95% confidence interval 1.41 to 5.57; P=0.003) (table 4 and supplementary table D). On multivariable analysis, both age under 1 month (odds ratio 3.21, 1.36 to 7.66; P=0.008) and age between 10 and 14 years (3.23, 1.55 to 6.99; P=0.002) were associated with admission to critical care (reference age group 15-19 years), but we found no association with sex on either univariable or multivariable analysis (table 3, table 4, and supplementary table D).

**Table 3:** Demographics stratified by admission to critical care. Values are numbers (percentages) unless stated otherwise

Variable	Standard ward admission (n=516; 81.6%)	Critical care admission (n=116; 18.4%)	P value*
Median (interquartile range) age, years	3.7 (0.3-13.8)	8.9 (0.4-13.5)	0.29
Neonate (<1 month):			0.007
No	482 (93.4)	99 (85.3)	
Yes	34 (6.6)	17 (14.7)	
Age group, years:			<0.001
<1	186 (36.0)	31 (26.7)	
1-4	90 (17.4)	15 (12.9)	
5-9	73 (14.1)	18 (15.5)	
10-14	58 (11.2)	33 (28.4)	
15-19	109 (21.1)	19 (16.4)	
Sex at birth:			0.300
Male	286 (55.4)	71 (61.2)	
Female	229 (44.4)	45 (38.8)	
Missing	1 (0.2)	0 (0.0)	
Ethnicity:			0.001
White	282 (54.7)	41 (35.3)	
Black	36 (7.0)	19 (16.4)	
South Asian	49 (9.5)	13 (11.2)	
Other	92 (17.8)	26 (22.4)	
Missing	57 (11.0)	17 (14.7)	
Admitted >5 days before symptom onset:			0.009
No	437 (84.7)	92 (79.3)	
Yes	36 (7.0)	18 (15.5)	
Missing	43 (8.3)	6 (5.2)	
Any comorbidity:			0.009
No/unknown	306 (59.3)	53 (45.7)	
Yes	210 (40.7)	63 (54.3)	
Prematurity†:			0.001
No	135 (26.2)	15 (12.9)	
Yes	30 (5.8)	15 (12.9)	
Missing	351 (68.0)	86 (74.1)	
Respiratory comorbidity:			0.019
No	470 (91.1)	103 (88.8)	
Yes	21 (4.1)	12 (10.3)	
Missing	25 (4.8)	1 (0.9)	
Cardiac comorbidity:			0.018
No	468 (90.7)	102 (87.9)	
Yes	25 (4.8)	13 (11.2)	
Missing	23 (4.5)	1 (0.9)	
Obesity:			0.028
No	477 (92.4)	108 (93.1)	
Yes	10 (1.9)	7 (6.0)	
Missing	29 (5.6)	1 (0.9)	
Immunosuppressant use before presentation‡:			1.00
No	443 (85.9)	101 (87.1)	
Yes	43 (8.3)	10 (8.6)	
Missing	30 (5.8)	5 (4.3)	

* Categorical variables analysed using Fisher’s exact test and continuous variables by Kruskal-Wallis test.

† Defined as birth before completion of 37 weeks’ gestation.

‡ Includes oral but not inhaled corticosteroids.

**Table 4:** Factors associated with admission to critical care unit. Values are numbers (percentages) unless stated otherwise

Variable	Standard ward admission (n=516; 81.6%)	Critical care admission (n=116; 18.4%)	Odds ratio (95% CI)	
			Univariable	Multivariable
Sex at birth:				
Male	286 (80.1)	71 (19.9)	-	-
Female	229 (83.6)	45 (16.4)	0.79 (0.52 to 1.19); P=0.266	0.82 (0.51 to 1.31); P=0.405
Age group:				
15-19 years	109 (85.2)	19 (14.8)	-	-
<1 month	34 (66.7)	17 (33.3)	2.87 (1.34 to 6.16); P=0.007	3.21 (1.36 to 7.66); P=0.008
1 month to <1 year	152 (91.6)	14 (8.4)	0.53 (0.25 to 1.09); P=0.088	0.53 (0.22 to 1.25); P=0.151
1-4 years	89 (85.6)	15 (14.4)	0.97 (0.46 to 2.01); P=0.928	1.28 (0.57 to 2.89); P=0.545
5-9 years	73 (80.2)	18 (19.8)	1.41 (0.69 to 2.89); P=0.338	1.33 (0.58 to 3.05); P=0.493
10-14 years	58 (63.7)	33 (36.3)	3.26 (1.72 to 6.33); P<0.001	3.23 (1.55 to 6.99); P=0.002
Ethnicity:				
White	281 (87.3)	41 (12.7)	-	-
Black	36 (65.5)	19 (34.5)	3.62 (1.88 to 6.86); P<0.001	2.82 (1.41 to 5.57); P=0.003
South Asian	49 (79.0)	13 (21.0)	1.82 (0.88 to 3.56); P=0.091	1.86 (0.87 to 3.77); P=0.094
Other	92 (78.0)	26 (22.0)	1.94 (1.11 to 3.32); P=0.017	1.91 (1.07 to 3.34); P=0.025
Any comorbidity:				
No/unknown	305 (85.2)	53 (14.8)	-	-
Yes	210 (76.9)	63 (23.1)	1.73 (1.15 to 2.60); P=0.008	1.42 (0.89 to 2.28); P=0.141

Univariable and multivariable logistic regression analyses used potential predictors identified a priori and during exploratory analyses.

On univariable analysis, children with comorbidities were more likely to be admitted to critical care than those without comorbidities (odds ratio 1.73, 1.15 to 2.60; P=0.008); however, this no longer reached significance in the multivariable model (odds ratio 1.42, 0.89 to 2.28; P=0.141). Comorbidities most commonly associated with critical care admission on univariable analysis were prematurity (50% (15/30) of critical care admissions *v* 18% (30/165) of standard care admissions; P=0.001), respiratory comorbidities (10% (12/115) *v* 4% (21/491); P=0.019), cardiac comorbidities (13/115 (11%) *v* 25/493 (5%); P=0.018), and obesity (6% (7/115) *v* 2% (10/487); P=0.028) (table 3 and supplementary table E). Children receiving critical care were more likely to have been admitted to hospital more than five days before their symptoms started (indicating likely hospital acquired infection) than those receiving ward level care (16% (18/110) *v* 8% (36/473); P=0.009). We found no association between previous immunosuppressant use and critical care admission (table 3).

Children admitted to critical care were more likely to have presented with diarrhoea, (38% (40/104) *v* 13% (58/448); P<0.001), conjunctivitis (16% (16/100) *v* 2% (10/412); P<0.001), and altered consciousness/confusion (19% (19/99) *v* 6% (24/434); P<0.001) than those cared for on a standard ward (supplementary table F). They were objectively more unwell at presentation than those receiving standard ward care (median PEWS of 5 (2.0-7.0) *v* 2 (1.0-4.0); P<0.001) (supplementary table G). We also observed significant differences in haematological, biochemical, and radiological abnormalities between the two groups at presentation (supplementary table H). In particular, children admitted to critical care had a lower platelet count (median 192.0×10^9^/L (interquartile range 133.0-280.5×10^9^/L) *v* 296.5×10^9^/L (229.0-383.5×10^9^/L); P<0.001), a higher neutrophil count (7.7×10^9^/L (4.3-12.3×10^9^/L) *v* 4.6×10^9^/L (2.3-8.2×10^9^/L); P<0.001), and higher C reactive protein (64.5 (11.1-200.2) mg/L *v* 11.0 (3.0-54.5) mg/L; P<0.001) at presentation than those cared for on a standard ward. Children admitted to critical care were also more likely to have infiltrates on a chest radiograph (58% (47/81) *v* 32% (52/162); P<0.001).

We did a sub-analysis for critical care admission excluding any children who met the criteria for MIS-C. In this subgroup, the associations described above persisted, although obesity was no longer significantly associated with critical care admission (supplementary table I). In patients without MIS-C, prematurity and respiratory and cardiac comorbidities remained significantly associated with admission to critical care in addition to neurological (22% (17/77) *v* 10% (46/481); P=0.003), neurodisability (13% (9/71) *v* 5% (22/463); P=0.014), and gastrointestinal (9% (7/77) *v* 2% (11/480); P=0.007) comorbidities (supplementary table J), as is generally the case for children and young people most at risk of admission to critical care. After exclusion of children with MIS-C, conjunctivitis and diarrhoea were no longer significantly associated with critical care admission (supplementary table K); instead, shortness of breath (44% (31/71) *v* 28% (123/445); P=0.008) was associated with critical care admission. Total PEWS at presentation remained associated with critical care admission (supplementary table L). As expected, when cases of MIS-C were excluded, blood tests in patients admitted to critical care showed less of an inflammatory pattern compared with those cared for on a standard ward (median neutrophils 6.2×10^9^/L (3.4-9.6×10^9^/L) in critical care admissions versus 4.5×10^9^/L (2.3-8.2×10^9^/L) in standard ward admissions (P=0.059) and median C reactive protein 21.4 (5.0-51.9) mg/L *v* 9.0 (3.0-46.9) mg/L (P=0.069)) (supplementary table M).

### Outcomes

Outcome data were available for 627 children (table 2). Six (1%) children and young people died in hospital. Three were neonates (age <28 days) with severe comorbidities/illness—very premature, complex congenital cardiac anomaly, and bacterial sepsis. Three were aged 15-18 years, two of whom had profound neurodisability with pre-existing respiratory compromise; the other was immunosuppressed by chemotherapy for malignancy and had bacterial sepsis. Two children under 5 years old, both with life limiting, complex comorbidities, were discharged with planned palliative care and cause of death was not related to covid-19. Eighty nine per cent (557/627) of children and young people were discharged alive, and 10% (62/627) continued to receive care at the date of reporting (table 2).

### Patients meeting WHO preliminary case definition for MIS-C

**Fig 3:**
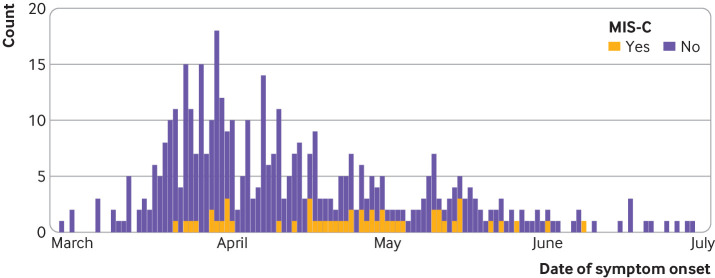
Dates of symptom onset for paediatric cases of SARS-CoV-2 infection and cases meeting WHO preliminary criteria for multisystem inflammatory syndrome in children and adolescents (MIS-C) in ISARIC WHO CCP-UK cohort over time

Eleven per cent (52/456) of children met the WHO preliminary case definition for MIS-C (table 5 and supplementary figure F).^9^ The first patient identified developed symptoms in mid-March, when covid-19 cases were increasing nationally, followed by a small number of cases identified steadily throughout the surveillance period (fig 3). Geographically, the highest number of children with MIS-C came from areas with the largest covid-19 outbreaks—namely, the Midlands and Greater London (supplementary figure G).

**Table 5:** Demographics at presentation and therapies administered stratified by multisystem inflammatory syndrome in children and adolescents (MIS-C) status. Values are numbers (percentage) unless stated otherwise

Variable	Did not meet MIS-C criteria (n=404; 88.6%)	Met MIS-C criteria (n=52; 11.4%)	P value*
Median (interquartile range) age, years	1.6 (0.2 to 12.9)	10.7 (8.3 to 14.1)	<0.001
Age group, years:			<0.001
<1	164 (40.6)	1 (1.9)	
1-4	70 (17.3)	4 (7.7)	
5-9	43 (10.6)	16 (30.8)	
10-14	48 (11.9)	22 (42.3)	
15-19	79 (19.6)	9 (17.3)	
Sex at birth:			1.00
Male	241 (59.7)	31 (59.6)	
Female	163 (40.3)	21 (40.4)	
Missing	0 (0.0)	0 (0.0)	
Ethnicity:			0.004
White	207 (51.2)	16 (30.8)	
Black	30 (7.4)	9 (17.3)	
South Asian	44 (10.9)	4 (7.7)	
Other	74 (18.3)	16 (30.8)	
Missing	49 (12.1)	7 (13.5)	
White ethnicity:			0.004
No	148 (36.6)	29 (55.8)	
Yes	207 (51.2)	16 (30.8)	
Missing	49 (12.1)	7 (13.5)	
Any comorbidity:			0.052
No/unknown	227 (56.2)	37 (71.2)	
Yes	177 (43.8)	15 (28.8)	
Obesity:			0.005
No	379 (93.8)	46 (88.5)	
Yes	6 (1.5)	5 (9.6)	
Missing	19 (4.7)	1 (1.9)	
Immunosuppressant use before presentation†:			0.60
No	348 (86.1)	48 (92.3)	
Yes	35 (8.7)	3 (5.8)	
Missing	21 (5.2)	1 (1.9)	
Admitted >5 days before symptom onset:			0.014
No	345 (85.4)	52 (100.0)	
Yes	35 (8.7)	0 (0.0)	
Missing	24 (5.9)	0 (0.0)	
Intravenous steroid:			<0.001
No	333 (82.4)	20 (38.5)	
Yes	20 (5.0)	24 (46.2)	
Missing	51 (12.6)	8 (15.4)	
High flow nasal cannula oxygen:			<0.001
No	343 (84.9)	29 (55.8)	
Yes	47 (11.6)	23 (44.2)	
Missing	14 (3.5)	0 (0.0)	
Non-invasive ventilation:			<0.001
No	366 (90.6)	34 (65.4)	
Yes	31 (7.7)	18 (34.6)	
Missing	7 (1.7)	0 (0.0)	
Invasive ventilation:			0.001
No	361 (89.4)	38 (73.1)	
Yes	36 (8.9)	14 (26.9)	
Missing	7 (1.7)	0 (0.0)	
Inotropic support:			<0.001
No	362 (89.6)	24 (46.2)	
Yes	18 (4.5)	25 (48.1)	
Missing	24 (5.9)	3 (5.8)	
ICU/HDU admission:			<0.001
No	342 (84.7)	14 (26.9)	
Yes	62 (15.3)	38 (73.1)	
Missing	0 (0.0)	0 (0.0)	
Outcome:			0.49
Died	4 (1.0)	0 (0.0)	
Discharged alive	353 (87.4)	43 (82.7)	
Ongoing care	37 (9.2)	8 (15.4)	
Palliative discharge	2 (0.5)	0 (0.0)	
Missing	8 (2.0)	1 (1.9)	

HDU=high dependency unit; ICU=intensive care unit.

* Categorical variables analysed using Fisher’s exact test and continuous variables by Kruskal-Wallis test.

† Includes oral but not inhaled corticosteroids.

Children who met the WHO preliminary definition for MIS-C were significantly older than those who did not (median age 10.7 (8.3-14.1) *v* 1.6 (0.2-12.9) years; P<0.001) and were significantly more likely to be of non-white ethnicity (64% (29/45) *v* 42% (148/355); P=0.004) (table 5). MIS-C was associated with obesity (10% (5/51) *v* 2% (6/385); P=0.005) but not with any other comorbidity (supplementary table N). No children with MIS-C were admitted more than five days before symptom onset (table 5). In addition to the WHO preliminary case definition features (fever, rash, conjunctivitis, and gastrointestinal symptoms), the children with MIS-C were also more likely to present with fatigue (51% (24/47) *v* 28% (86/302); P=0.004), headache (34% (16/47) *v* 10% (26/263); P<0.001), myalgia (34% (15/44) *v* 8% (21/270); P<0.001), sore throat (30% (14/47) *v* (12% (34/284); P=0.003), and lymphadenopathy (20% (9/46) *v* 3% (10/318); P<0.001) than were children who did not meet MIS-C criteria (supplementary table O). Children with MIS-C also had a higher PEWS at presentation (median 5.0 (2.8-6.2) *v* 3.0 (1.0-5.0); P<0.001) and were more likely to have reduced consciousness (25% (13/51) *v* 9% (33/350); P=0.001) than those who did not meet the criteria (supplementary table P and supplementary figure H).

Children with MIS-C were five times more likely than others to be admitted to critical care (73% (38/52) *v* 15% (62/404); P<0.001) (table 5). They were more likely to receive intravenous corticosteroids (55% (24/44) *v* 6% (20/353); P<0.001), non-invasive (35% (18/52) *v* 8% (31/397); P<0.001) and invasive ventilation (27% (14/52) *v* 9% (36/397); P=0.001), and inotropic support (51% (25/49) *v* 5% (18/380); P=0.001) (table 5). Sixty five per cent (28/43) of patients with MIS-C received intravenous immunoglobulins, and 17% (7/42) received immunomodulatory therapy (three received anakinra, one tocilizumab, one adalimumab, one infliximab, and one unspecified) (supplementary table R). Fifty seven per cent (21/37) of MIS-C cases had one or more documented cardiac complications. Of these, 10 had impaired cardiac function on echocardiogram, nine had a pericardial effusion, three had electrocardiographic changes (heart block, junctional rhythm and T wave inversion, borderline ST elevation and changes of pericarditis), three had coronary artery dilatation, two had coronary artery aneurysm, two had myocarditis and two had valvular regurgitation. No deaths occurred in the MIS-C group.

Review of laboratory investigations found that children who met the criteria for MIS-C were more likely to have a platelet count of less than 150×10^9^/L than those who did not (32% (16/50) *v* 11% (38/348); P<0.001) (supplementary table Q). Children who met the MIS-C criteria also had lower lymphocyte counts (median 0.9×10^9^/L (0.7-1.7×10^9^/L) *v* 2.2×10^9^/L (1.3-3.8×10^9^/L); P<0.001) but higher neutrophil counts (8.3×10^9^/L (5.9-12.4×10^9^/L) *v* 4.6×10^9^/L (2.3-8.6×10^9^/L); P<0.001) and higher creatinine (55.0 (35.8-82.5) μmol/L *v* 30.0 (20.0-51.8) μmol/L; P<0.001) than those without (supplementary table Q).

Of the 52 patients with MIS-C, 56% (28/50) were polymerase chain reaction positive for SARS-CoV-2 (acute infection), 44% (22/50) were SARS-CoV-2 antibody positive (post-acute), and two were confirmed but the method was not specified. (supplementary table R). Patients with MIS-C who were antibody positive were younger (median age 10.0 (7.7-13.2) years *v* 12.4 (8.9-15.3) years; P=0.057) and more likely to be of non-white ethnicity (90% (19/21) *v* 45% (10/22); P=0.003) than those who were polymerase chain reaction positive. Although obesity had been associated with MIS-C (table 5), when analysed by SARS-CoV-2 detection method this feature seemed to be driven by those with MIS-C who were polymerase chain reaction positive, as none of the antibody positive patients were obese (19% (5/27) *v* 0% (0/22); P=0.056) (supplementary table S).

On comparing presenting symptoms, we found that conjunctivitis (71% (15/21) *v* 16% (4/25); P<0.001) and abdominal pain (95% (20/21) *v* 44% (12/27); P<0.001) were more common in patients who were in the antibody positive group, whereas those in the acute phase of infection and positive by polymerase chain reaction were more likely to present with shortness of breath (52% (14/27) *v* 14% (3/21); P=0.014) (supplementary table T).

Although PEWS was similar between the groups, children who were polymerase chain reaction positive were more likely to have a need for oxygen at presentation (40% (10/25) *v* 14% (3/22); P=0.056) (supplementary table U). Blood results at presentation were similar between the two groups, with the exception of prothrombin time, which was longer in the group who were polymerase chain reaction positive (median 14.8 (13.4-15.8) sec *v* 11.6 (11.0-12.0) sec; P=0.013) (supplementary table V).

Treatments received also differed between the two groups, with patients who were antibody positive being more likely to receive intravenous steroids (84% (16/19) *v* 35% (8/23); P=0.002) and intravenous immunoglobulin (86% (19/22) *v* 43% (9/21); P=0.004) than those who were polymerase chain reaction positive (supplementary table R). The need for critical care or respiratory or cardiovascular support did not differ between the two groups. Although cardiac complications were seen in both groups, they were more frequent in patients with MIS-C who were antibody positive (75% (15/20) *v* 35% (6/17); P=0.022) (supplementary table R).

## Discussion

Six hundred and fifty one children and young people aged under 19 years with laboratory confirmed SARS-CoV-2 were recruited to the ISARIC WHO CCP-UK study between 17 January and 3 July 2020, accounting for 0.9% of all patients in the whole cohort at that time. The median age of children with covid-19 was 4.6 (interquartile range 0.3–13.7) years. The cohort was predominantly male (56%) and of white ethnicity (57%), with most (58%) children having no known comorbidities. The most common presenting symptoms were fever, cough, shortness of breath, nausea, and vomiting, and a systemic mucocutaneous-enteric cluster of symptoms was seen. Eighteen per cent of children admitted to hospital needed critical care. Critical care admission was associated with age younger than 1 month, age 10-14 years, and black ethnicity. The all cause in-hospital case fatality rate for children and young people was strikingly low at 1% (6/627), compared with 27% (18 803/69 516) in the whole cohort of all ages (0-106 years) over the same time period. In this paediatric cohort, 11% of children and young people met the WHO preliminary criteria for MIS-C, which was associated with older age, non-white ethnicity, and admission to critical care. MIS-C cases were first identified in mid-March when cases of covid-19 began to rise in the UK. In addition to the clinical criteria provide by WHO,^9^ we found that children with MIS-C were more likely to present with fatigue, headache, myalgia, sore throat, and lymphadenopathy, as well as a lower platelet count than children with SARS-CoV-2 who did not meet the MIS-C criteria.

Children who had been admitted to hospital for more than five days before symptom onset were also more likely to be admitted to critical care. By definition, this group includes children with comorbidity, which was associated with critical care admission. SARS-CoV-2 nosocomial infections in children are not well reported, and this area warrants closer scrutiny, ideally with the use of viral sequence data.

This study identified children meeting the criteria for MIS-C in both the acute phase of infection (polymerase chain reaction positive) and post-acute or convalescent phase of infection (antibody positive) groups. Although the two groups shared many similarities, important differences included the post-acute group being more strongly associated with non-white ethnicity and muco-enteric symptom presentation (abdominal pain and conjunctivitis), whereas the acute group presented more commonly with respiratory symptoms. Cardiac complications occurred across both groups but were more common in the post-acute patients, who were also more likely to receive intravenous steroids and immunoglobulins.

### Strengths and limitations of study

This study is unique in that data for patients with laboratory confirmed covid-19 were collected prospectively and throughout the admission. The ISARIC WHO CCP-UK study had previously been activated in 2016 and 2018 for cases of Middle East respiratory syndrome (MERS) and monkeypox, and so was prepared for the SARS-CoV-2 pandemic, allowing swift activation. Consequently, in addition to reporting the clinical characteristics, risk factors, and outcomes of covid-19 in children, this dataset provided a unique opportunity to objectively monitor the emergence and progression of a novel multisystem inflammatory syndrome in the UK, while minimising recall bias. The first patient meeting the criteria for MIS-C was identified on 20 March 2020, and the first published cases were reported on 6 May 2020.^8^ Comparison with overall covid-19 cases confirms the sporadic occurrence of MIS-C throughout the first peak of the covid-19 pandemic in the UK. In contrast to previous reports, our analysis was limited to children admitted with laboratory confirmed SARS-CoV-2, which allowed us to clearly define the picture of covid-19 in children and reduce confounding by other potential causes.

The ISARIC WHO CCP-UK database was estimated to represent two thirds of hospital admissions for covid-19 across England, Wales, and Scotland at the time of extraction. It is therefore susceptible to selection bias, particularly as tertiary centres with critical care units and specialist children’s hospitals are more likely to have dedicated research teams, potentially skewing the severity and age of the patients reported. The most common presenting symptoms in children in our study (fever, cough, and dyspnoea) reflect the original case definition for SARS-CoV-2 testing in the UK, suggesting that this paediatric cohort is likely to have been influenced by the testing criteria.

The PEWS is validated up to 16 years of age.^14^ As ranges of clinical observations do not vary much between 16 and 18 year olds,^20^ PEWS scoring was extended to all those under 19 years. To identify children and young people meeting the WHO criteria for MIS-C, data on C reactive protein and fever are needed. Decisions to measure C reactive protein and other parameters were at physicians’ discretion. Children missing either of these variables were excluded from this analysis.

A limitation of this study is the use of a case record form that was agnostic to age and so not specifically tailored for paediatric data collection, particularly regarding comorbidities. Some of this information was available in free text, but these data were incomplete. By design, we were not able to differentiate between people whose symptoms were directly attributable to SARS-CoV-2 infection and those who had been admitted for other reasons and then found to be positive for the virus. The study relied primarily on polymerase chain reaction testing as evidence of SARS-CoV-2 infection, as diagnostic serology was not available at the start of the pandemic. This could have limited early recruitment of MIS-C cases. Finally, in order to share findings from this study promptly as an urgent public health research priority, these analyses were performed on a cohort with ongoing data collection and missing data, the proportion of which will decrease with time. This may affect our estimate of the incidence of MIS-C at 11% (52/456) among children and young people with proven SARS-CoV-2 infection, which is calculated on the 70% (456/651) who had data available for fever and C reactive protein, thus allowing WHO preliminary criteria to be applied. Children without features of MIS-C or sepsis are not likely to have their clotting and inflammatory markers measured, so reducing the pool of complete data and the denominator. We do not have data for children identified as infected with SARS-CoV-2 in the community who were not admitted to hospital, and we cannot yet report on sequelae of covid-19 in children after discharge.

### Comparison with other studies

Children and young people aged under 19 years accounted for 0.9% (651/69 516) of the ISARIC WHO CCP-UK cohort on 3 July 2020, which is broadly consistent with 2% reported in China and 1.7% in North America.^1^ ^2^ Our cohort of paediatric patients admitted to hospital had a median age of 4.6 years, which was similar to an Italian cohort (3.3 years^21^), but younger than Chinese (6-7 years^5^ ^6^) and North American (11 years^2^) cohorts; however, these other cohorts were not limited to children admitted to hospital. Although respiratory presentations were most common, 35% of children also had gastrointestinal symptoms at presentation, which is higher than the 10-22% reported in other paediatric literature.^2^ ^21^ ^22^ Gastrointestinal symptoms have also been prominent in children presenting with infection by MERS-CoV (28%) and severe acute respiratory syndrome coronavirus (SARS-CoV) (30%).^23^ ^24^ We also identified a distinct systemic mucocutaneous-enteric cluster of symptoms in the acute phase of SARS-CoV-2 infection, which shows overlap with the WHO preliminary case definition for MIS-C.

Children of black ethnicity were over-represented, comprising 10% of our paediatric cohort compared with a population representation of 4.7% of all children under 18 years across England and Wales and 1% in Scotland.^25^ ^26^ This finding may also be influenced by the ethnic composition of the population served by the sites recruiting to this study. Black ethnicity was also associated with increased odds of admission to critical care on multivariable analysis, consistent with reports for adult populations suggesting that South Asian and black ethnicities are disproportionately severely affected by SARS-CoV-2 infection.^27^ ^28^ ^29^ Studies of paediatric covid-19 from other countries either have been from ethnically homogenous groups or have not reported ethnicity, making comparisons difficult.

The rate of admission to critical care in our cohort was 18%, compared with 10% reported in a North American cohort of children admitted to hospital and 13% in a multicentre cohort study across 25 European countries.^2^ ^22^ As previously noted, this rate may be elevated in our study owing to hospitals with dedicated paediatric research teams being more likely to provide paediatric critical care. The prevalence of comorbidities (54%) in children admitted to critical care in our cohort was also similar to that reported in the European multicentre study (52%).^22^ Obesity was associated with critical care admission in our paediatric cohort, in agreement with adult data from ISARIC WHO CCP-UK.^13^ In England, 20% of children are obese by 11 years of age.^30^ Childhood obesity, however, is also influenced by deprivation,^30^ which we did not analyse in our study. Age under 1 month was associated with increased odds of critical care admission, in agreement with the European cohort.^22^ Thirty five per cent of children in our study were under 1 year old, which may reflect a low threshold for admissions of infants by clinicians rather than severe concerns about their clinical condition. This may explain the predominance of younger children in our study. In addition, the association between age under 1 month and admission to critical care might be explained if these babies were already admitted to neonatal intensive care and undergoing regular SARS-CoV-2 screening.

Using adapted WHO criteria,^9^ we identified 52 patients meeting the criteria for multisystem inflammatory syndrome. Initial UK reports described children admitted to hospital with circulatory shock and a hyperinflammatory state with features similar to toxic shock or Kawasaki disease.^8^ Children fulfilling the case definition for MIS-C have been reported in multiple regions experiencing large outbreaks of covid-19, including England (UK),^31^ Paris (France),^11^ Bergamo (Italy),^10^ and New York City (USA).^32^ Ours is the first report, however, to identify cases and timelines by using a prospective national data collection strategy. MIS-C seems to be temporally associated with covid-19, but a causal relation remains to be established. Older age and non-white ethnicity were associated with MIS-C in our study, in agreement with a recent case series of 99 children with MIS-C from New York State (USA), where 63% were of non-white ethnicity and 69% were aged between 6 and 20 years.^32^ Children in our study with MIS-C were much more unwell than other children with covid-19, with 51% needing inotropic support, compared with 20% in the Italian cohort,^10^ 47% in the French cohort,^11^ and 62% in the New York cohort.^29^

It is becoming apparent that MIS-C can present both in children with acute SARS-CoV-2 infection and in the post-acute or convalescent phase of infection. A large case series of 565 children with MIS-C across North America has been recently reported by the Centers for Disease Prevention and Control (CDC), which used latent class analysis to identify three classes of MIS-C.^33^ Class 1 predominantly comprises patients who were antibody positive and polymerase chain reaction negative for SARS-CoV-2, with multiple systems involved, a strong association with cardiac complications, and a greater likelihood of having received intravenous immunoglobin and corticosteroids. These class 1 patients resemble the post-acute group in our analysis. The CDC describes class 2 as children who were predominantly polymerase chain reaction positive with more respiratory involvement, and these resemble the acute group in our analysis.

We believe that the characterisation of SARS-CoV-2 illness in children into polymerase chain reaction positive versus serology positive as distinct entities is likely to be an oversimplification. Instead, our data suggest that clinical presentation varies at different time points in the course of SARS-CoV-2 infection and immune response.

Across the whole of our paediatric cohort, we identified a distinct cluster presenting with systemic mucocutaneous-enteric symptoms (rash, conjunctivitis, diarrhoea, vomiting, and abdominal pain) in addition to headache, myalgia, sore throat, fatigue, and lymphadenopathy, which overlapped closely with the WHO preliminary case definition.^9^

MIS-C can present in both the acute and convalescent phases of SARS-CoV-2 infection. The significant associations between MIS-C and fatigue, headache, myalgia, sore throat, and lymphadenopathy in our cohort may be useful in refining the case definition. In addition, the association of MIS-C with platelet count less than 150×10^9^/L and low lymphocyte counts agrees with previous reports.^31^ ^32^ These important findings may assist in differentiating this syndrome from other illnesses, particularly Kawasaki disease in which platelet counts are typically elevated.

### Conclusion and policy implications

Our data confirm less severe covid-19 in children and young people with SARS-CoV-2 infection than in adults. Admission to critical care was associated with age under 1 month, age 10-14 years, and black ethnicity. In agreement with previous reports, we found older age and non-white ethnicity to be associated with MIS-C.

We also report that patients with MIS-C who are antibody positive for SARS-CoV-2 are more likely to be of non-white ethnicity, have mucocutaneous-enteric symptoms and cardiac complications, and have received intravenous immunoglobulins and corticosteroids than those who are in the acute phase of infection (polymerase chain reaction positive).

We have identified a systemic mucocutaneous-enteric symptom cluster across the whole cohort. In addition, we have provided evidence for refining the WHO case definition for MIS-C, including an association with low platelet count, fatigue, headache, myalgia, sore throat, and lymphadenopathy.

**What is already known on this topic**

Less information on ethnicity, comorbidities, and clinical and laboratory findings is available in children with SARS-CoV-2 than in adults
A multisystem inflammatory syndrome in children and adolescents (MIS-C) temporally associated with SARS-CoV-2 has been widely reported
However, all reports to date arise from retrospective cases series that are vulnerable to ascertainment, selection, and recall bias

**What this study adds**

Severe disease was rare and death exceptionally rare in this is a large prospective cohort study of children admitted to hospital with laboratory confirmed covid-19
Ethnicity seems to be a factor in both critical care admission and MIS-C
This cohort has enabled identification of additional clinical and laboratory characteristics that should help to refine the WHO criteria for MIS-C

## Web extra

Extra material supplied by authors

Web appendix: Supplementary material

## Notes

Contributors: OVS, KAH, LS, PLO, JSN-V-T, PWH, LM, GC, WJD, PJMO, JKB, EMH, ABD, and MGS developed the concept of the study. SH, MG, LM, EMH, and ABD were responsible for data curation. OVS, LPo, CF, TMD, SS, CE, MGP, SL, IPS, EMH, ABD, and MGS analysed the data. JSN-V-T, PWH, GC, PJMO, JKB, ABD, and MGS obtained funding. OVS, KAH, LT, LP, SL, EMH, and ABD were involved in investigation. OVS, KAH, LT, LPo, SL, EMH, ABD, and MGS developed the methodology. KAH, LT, HH, SH, MG, CD, LPa, LS, and LM were responsible for administration. LS, PO, PWH, LM, and MGS coordinated resources. OVS, CF, TMD, SS, CE, and LM developed software. HH, SL, PO, JSN-V-T, LM, PJMO, JKB, ABD, and MGS provided supervision. KAH, LT, and LPo validated the data. OVS, CF, TMD, SS, CE, MGP, EMH, and ABD were involved in data visualisation. OVS, KAH, LT, LPo, SL, EMH, ABD, and MGS wrote the original draft of the manuscript. OVS, KAH, LT, LPo, CF, TMD, MGP, LPa, SL, IPS, JSN-V-T, JKB, EMH, ABD, and MGS reviewed and edited the manuscript. The corresponding author attests that all listed authors meet authorship criteria and that no others meeting the criteria have been omitted. MGS is the guarantor.

Ethical approval: Ethical approval was given by the South Central - Oxford C Research Ethics Committee in England (ref 13/SC/0149), the Scotland A Research Ethics Committee (ref 20/SS/0028), and the WHO Ethics Review Committee (RPC571 and RPC572, 25 April 2013).

Data sharing: The Independent Data and Material Access Committee welcomes applications for access to data and materials (https://isaric4c.net).

Transparency: The lead author (the manuscript's guarantor) affirms that the manuscript is an honest, accurate, and transparent account of the study being reported; that no important aspects of the study have been omitted; and that any discrepancies from the study as planned (and, if relevant, registered) have been explained.

Dissemination to participants and related patient and public communities: ISARIC4C has a public facing website and twitter account (@CCPUKstudy). We are engaging with print and internet press, television, radio, news, and documentary programme makers. We will explore distribution of findings with The Asthma UK and British Lung Foundation Partnership and take advice from NIHR Involve and GenerationR Alliance Young People’s Advisory Groups.

Provenance and peer review: Not commissioned; externally peer reviewed.
