# Supplementary material for: Clinical characteristics of children and young people admitted to hospital with covid-19 in United Kingdom: prospective multicentre observational cohort study
Source: BMJ. 2020 Aug 27;370:m3249. doi: 10.1136/bmj.m3249 (PMC7488201; doi:10.1136/bmj.m3249)
Supplement: Supplementary file 1 — Web appendix: Supplementary material [file swao060426.ww1.pdf]

Clinical characteristics of children and young people hospitalised with covid-19 in the United Kingdom using the ISARIC WHO Clinical Characterisation Protocol: prospective multicentre observational cohort study

Swann OV, Holden, KA, Turtle L et al.

Supplementary Methods

Readmissions / transfers

NHS / CHI numbers were reviewed for the cohort to allow identification of children who were included more than once. These admissions were reviewed. If the duplicate represented a readmission, only the first admission was included in the final database. The exception to this was if the second admission resulted in higher level care (i.e. critical care admission), in which case that admission was retained instead. When duplicate entries were the result of transfers, the admission with highest level of care was retained in the database. In this case, features of the illness at the time of the earliest presentation (observations, blood tests, symptoms) were incorporated into that admission, in order to reflect the earliest state of health of the child during that illness.

WHO preliminary case definition for MIS-C, coagulation criteria

Prothrombin time (PT) normal reference ranges are age, reagent and analyser-specific (1). Since local reference ranges were unknown, a PT value >14 seconds for term neonates and older children and >16 seconds in preterm neonates was considered above the upper limit of normal based on published neonatal and paediatric cohorts (2–4).

Recoding of paediatric comorbidity variables:

Free text comorbidity information and review of medications on admission was used to enrich and recode children into comorbidity categories more relevant to the paediatric population. Two paediatric clinical reviewers (Authors OVS and LP) matched free-text comorbidity descriptions for all included children against case report form (CRF) coded comorbidities, and created new paediatric categories based on commonly occurring paediatric comorbidities not coded for in the CRF. Revised paediatric comorbidity coding included the generation of categories for genetic, metabolic, neurodisability and other endocrine (excluding diabetes) comorbidities. This free-text review process also highlighted that children had been inconsistently coded into the original haematological and

oncological variables, requiring the creation of a combined category for haematological / oncological / immunological diagnoses.

In a complementary process, recorded medications on admission were reviewed for all included children. Participants were systematically searched for free text entries of medications, or combinations of medications, associated with specific paediatric conditions. For example, inhaled corticosteroids or long-acting B2 agonists were considered to imply a diagnosis of asthma unless an alternative respiratory condition was recorded. Nebulised colomycin was considered to imply a chronic respiratory condition. One child was receiving Orkambi, specific to Cystic Fibrosis. The combination of baclofen and hyoscine was considered to imply neurodisability. Similar searches were undertaken for insulins, anti-convulsant, anti-hypertensive and cardiac drugs. Drug combinations were compared against free-text and coded comorbidities, and conditions recoded were necessary. Searches were also undertaken for any immunosuppressive or immunomodulatory drugs recorded on admission. Results were compared against coding for presence of immunosuppressive drugs and recoded where necessary.

Symptom clustering analysis:

Hierarchical clustering was used to partition the symptoms in groups such that symptoms within a group tend to appear together. This was performed using agglomerative hierarchical clustering, a 'bottom-up' approach, that starts by assigning each symptom to its own group, and then successively merging pair of groups that are closest to each other (5). 'Complete linkage' clustering was used where the distance between two groups is defined as the maximum distance between a pair of symptoms belonging to one group each. The distance between two symptoms was characterised by Jaccard distance where Jaccard distance is calculated by subtracting the Jaccard index from 1 (6). Jaccard index is a measure of similarity that computes the ratio of the number of times two symptoms appear together in the data and the number of times either of them appears in the data. The index varies between 0 and 1 with 0 implying that the two symptoms never appear together (no co-occurrence), and 1 implying that the two symptoms only appear together (co-occurrence only). The result of hierarchical clustering was summarised using dendrogram which shows how the groups were merged together starting from individual symptoms, and the relative distances among these groups (shown as height). A threshold can be applied on the distance between groups to choose a specific number of clusters. The optimal number of clusters was chosen using average silhouette index which measures the separation among clusters, i.e., a higher average silhouette index is better (7). The optimal number of clusters were found to be 4 (see Figure A) for

20 symptoms. We included symptoms with >2% prevalence across the cohort, and therefore, bleeding (1.69%, 11/651) and ear pain (1.23%, 8/651) were excluded from the analysis. Bootstrapping was performed to assess the stability of the clustering, i.e., the generation of 1000 new datasets of the same size by resampling the original dataset with replacement. The optimal number of clusters was computed in the resampled data and 4 clusters was observed to be most frequent (see Figure B). The cluster assignment of a pair of symptoms was checked over 1000 bootstraps assuming there are 4 clusters in each and observed that the same pair of symptoms are usually put in the same cluster random samples (see Figure C). The average adjusted Rand index between cluster assignments on the original data and that in a bootstrap is 0.56 (where adjusted Rand index of 0 implies that the two cluster assignments are independent, and 1 implies that they are the same (8)). The clustering of the original data is presented in Figure 1 in the main text. These analyses were performed in R using packages dendextend, mclust and gplots.

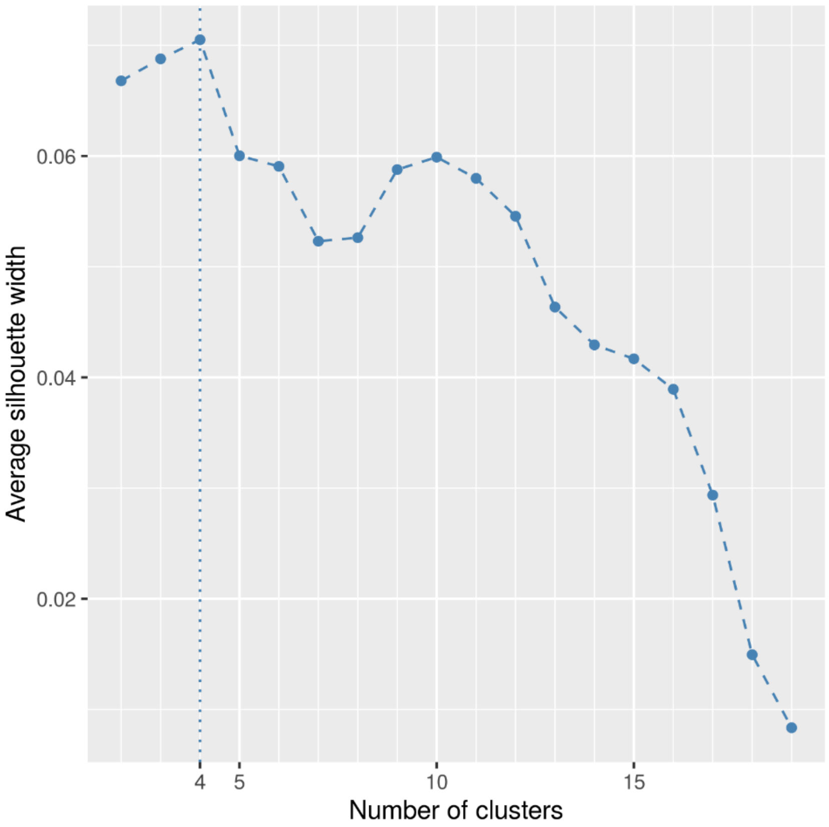

Figure A: *Variation of average silhouette index over number of clusters on the original data.*

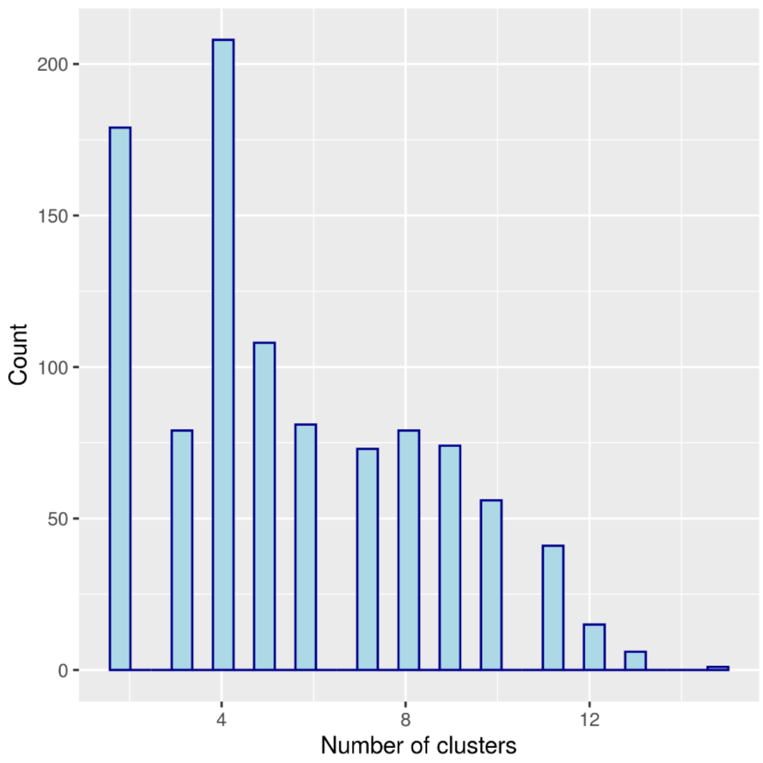

Figure B: *Histogram computing the optimal number of clusters over 1000 bootstraps.*

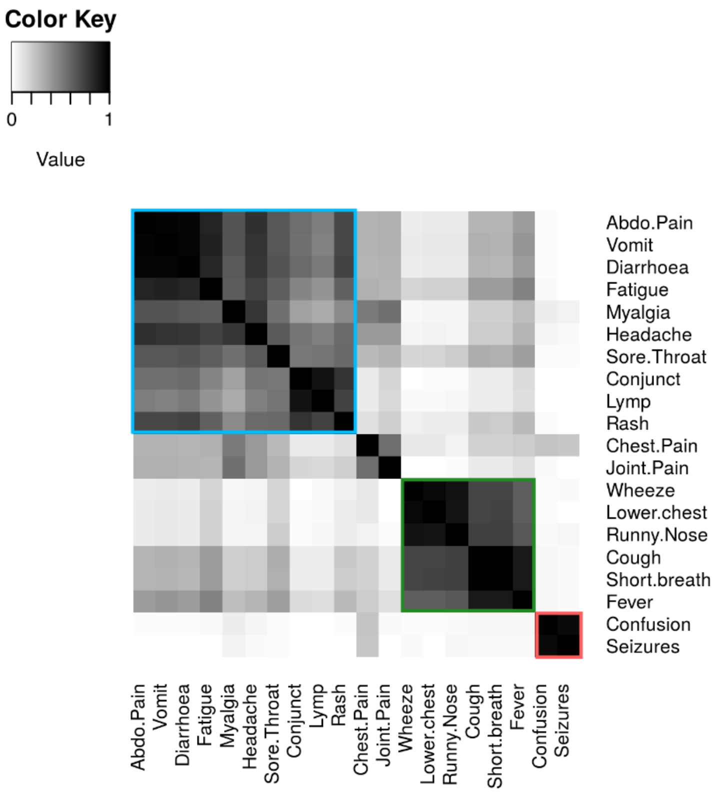

Figure C: *The figure shows the percentage of times a pair of symptoms have been put in the same cluster as the original data over 1000 bootstraps assuming that there are 4 clusters. Blue, green and red clusters correspond to those in Figure 1 in the main text. The fourth cluster (chest pain and joint pain) was a lose association.*

Symptoms by age group

We plotted symptoms on admission against age (in 5-year bands) and used chi-square tests for trend to investigate how these varied. We grouped symptoms according to two sets of clinical case definitions based on those in use in the United Kingdom (9): fever with at least one of cough, nasal discharge or congestion, shortness of breath, sore throat, or wheezing; and either cough or fever.

Further information on MIS-C cases:

Information on intravenous immunoglobulin use and immunomodulatory therapy was not routinely collected using the CRF. As such, when patients met the criteria for MIS-C, the recruiting centre was contacted directly to request this information. As such, these variables were only available for children who met the MIS-C criteria and not across the whole cohort.

Supplementary results

Co-infections in Multisystem Inflammatory Syndrome in Children and Adolescents (MIS-C)

Available data on tests for other infectious diseases were reviewed for the children meeting the Multisystem Inflammatory Syndrome in Children and Adolescents (MIS-C) and those with significant growth on blood or cerebrospinal fluid culture were excluded.

The following positive results were documented in the 52 remaining children:

- Five children - Epstein-Barr virus (EBV) PCR positive (blood)
- One child - *Escherichia coli* on urine culture
- One child - *Mycoplasma pneumoniae* and hepatitis B antibody positive (unspecified)
- One child - *Acinetobacter baumannii* (nasal / throat swab), antistreptolysin O and anti-DNAse B positive (blood)
- One child - Adenovirus PCR positive and parainfluenza PCR equivocal (nasopharyngeal aspirate (NPA))
- One child - *Candida albicans* (sputum), cytomegalovirus (CMV) PCR positive on bronchoalveolar lavage (BAL), adenovirus and coronavirus HKU1 PCR positive (NPA)
- One child - EBV PCR positive (blood and throat swab), CMV PCR positive (NPA and blood), HSV1 PCR (throat swab) and sapovirus PCR positive (stool)
- One child - *M. pneumoniae* PCR positive (NPA), EBV PCR positive (blood)
- One child - Parainfluenza type 2 PCR positive (NPA)
- One child - Sapovirus PCR equivocal (stool), adenovirus PCR positive (NPA), *Stenotrophomonas maltophilia* (stool)

Supplementary Tables

Supp Table A. *Comorbidities within the whole cohort. Prematurity defined as gestation < 37 weeks. Prematurity data was only routinely collected for children under 1 year old.*

Comorbidity		n (%)
Total N (%)		651 (100.0)
Prematurity	No	155 (23.8)
	Yes	46 (7.1)
	(Missing)	450 (69.1)
Neurological	No	549 (84.3)
	Yes	65 (10.0)
	(Missing)	37 (5.7)
Neurodisability	No	557 (85.6)
	Yes	32 (4.9)
	(Missing)	62 (9.5)
Respiratory	No	575 (88.3)
	Yes	35 (5.4)
	(Missing)	41 (6.3)
Asthma	No	570 (87.6)
	Yes	45 (6.9)
	(Missing)	36 (5.5)
Cardiac	No	573 (88.0)
	Yes	39 (6.0)
	(Missing)	39 (6.0)
Gastrointestinal	No	593 (91.1)
	Yes	20 (3.1)
	(Missing)	38 (5.8)
Haematology / Oncology / Immunology	No	567 (87.1)
	Yes	48 (7.4)
	(Missing)	36 (5.5)
Obesity	No	589 (90.5)
	Yes	17 (2.6)
	(Missing)	45 (6.9)
Malnutrition	No	602 (92.5)
	Yes	6 (0.9)
	(Missing)	43 (6.6)
Genetic	No	574 (88.2)
	Yes	23 (3.5)
	(Missing)	54 (8.3)
Other endocrine	No	587 (90.2)
	Yes	10 (1.5)
	(Missing)	54 (8.3)
Diabetes	No	598 (91.9)
	Yes	16 (2.5)
	(Missing)	37 (5.7)
Renal	No	599 (92.0)

	Yes	16 (2.5)
	(Missing)	36 (5.5)
Metabolic	No	595 (91.4)
	Yes	2 (0.3)
	(Missing)	54 (8.3)
Rheumatology	No	609 (93.5)
	Yes	4 (0.6)
	(Missing)	38 (5.8)
Other	No	582 (89.4)
	Yes	15 (2.3)
	(Missing)	54 (8.3)

15 children had comorbidities which did not meet any of the above categories. These included 3 recent operations (Ear Nose and Throat and abdominal), 2 dermatological diagnoses, 2 psychiatric illnesses, 1 renal transplant, 1 isolated spinal abnormality, 1 tuberculosis, 1 drug overdose, 1 undergoing cardiac investigation, 1 congenital abnormality, 1 hypocalcaemia of unknown cause and 1 unspecified.

Supp Table B. *Clinical observations and Paediatric Early Waring Score (PEWS) at presentation across the whole cohort, RR = respiratory rate, SpO_2 = oxygen saturations, HR = heart rate, BP = blood pressure. Clinical observations at presentation were not documented for 28 children, so PEWS could not be calculated.*

PEWS component		n (%)
Total N (%)		651 (100.0)
Total PEWS	Median (IQR)	3.0 (1.0 to 5.0)
PEWS RR	0	403 (61.9)
	1	149 (22.9)
	3	41 (6.3)
	(Missing)	58 (8.9)
PEWS $Sp0_2$	0	544 (83.6)
	1	23 (3.5)
	3	24 (3.7)
	(Missing)	60 (9.2)
PEWS O_2 requirement	0	505 (77.6)
	1	80 (12.3)
	(Missing)	66 (10.1)
PEWS HR	0	315 (48.4)
	1	131 (20.1)
	3	148 (22.7)
	(Missing)	57 (8.8)
PEWS Systolic BP	0	166 (25.5)
	1	165 (25.3)
	3	72 (11.1)
	(Missing)	248 (38.1)
PEWS Conscious level	0	496 (76.2)
	3	55 (8.4)
	(Missing)	100 (15.4)
PEWS Capillary refill time	0	185 (28.4)
	1	115 (17.7)
	(Missing)	351 (53.9)
PEWS Temperature	0	387 (59.4)
	1	207 (31.8)
	3	4 (0.6)
	(Missing)	53 (8.1)

Supp Table C. *Blood results on presentation. WBC = white blood cell, APTT = activated partial thromboplastin time, PT = prothrombin time, INR -= international normalised ration, ALT = alanine transaminase, AST = aspartate aminotransferase, CRP = C-reactive protein, CXR = chest X-ray.*

Investigation	Total n		n (%)
Total N (%)			651 (100.0)
Haemoglobin (g/L)	456	Median (IQR)	118.0 (105.0 to 133.0)
Haematocrit (%)	179	Median (IQR)	34.0 (30.7 to 39.0)
WBC ($x10^9$/L)	454	Median (IQR)	9.5 (6.4 to 13.8)
Lymphocyte count ($x10^9$/L)	451	Median (IQR)	2.0 (1.1 to 3.6)
Neutrophil count ($x10^9$/L)	452	Median (IQR)	5.2 (2.6 to 8.8)
Platelet count ($x10^9$/L)	450	Median (IQR)	275.5 (194.0 to 367.0)
APTT (sec)	133	Median (IQR)	31.7 (28.0 to 36.1)
PT (sec)	122	Median (IQR)	13.1 (11.9 to 14.6)
INR	43	Median (IQR)	1.1 (1.0 to 1.3)
ALT (IU/L)	303	Median (IQR)	24.0 (16.0 to 36.0)
AST (IU/L)	66	Median (IQR)	40.5 (26.5 to 70.0)
Bilirubin (µmol/L)	346	Median (IQR)	7.0 (5.0 to 15.0)
Glucose (mmol/L)	181	Median (IQR)	5.6 (4.8 to 6.8)
Urea (mmol/L)	402	Median (IQR)	3.5 (2.6 to 4.7)
Creatinine (µmol/L)	449	Median (IQR)	33.0 (21.0 to 56.0)
Sodium (mmol/L)	466	Median (IQR)	137.0 (135.0 to 139.0)
Potassium (mmol/L)	452	Median (IQR)	4.3 (3.9 to 4.9)
Lactate (mmol/L)	143	Median (IQR)	1.7 (1.1 to 2.7)
CRP (mg/L)	409	Median (IQR)	15.0 (4.0 to 69.0)
Infiltrates on any CXR	243	No	144 (59.3)
		Yes	99 (40.7)

Supp Table D. *Factors associated with admission to critical care unit. Univariable and multivariable logistic regression analyses were performed using potential predictors identified a priori and during exploratory analyses. Results are odds ratios with 95% confidence intervals. Data are otherwise n (%). OR, odds ratio; m, month; yr, year; OR, odds ratio with 95% confidence intervals, aOR, adjusted odds ratio.*

		Standard ward admission	Critical care admission	OR (univariable)	aOR (age, sex, adjusted multivariable)	aOR (age, sex and ethnicity adjusted multivariable)	aOR (age, sex and comorbidity adjusted multivariable)	aOR (full model multivariable)
Sex at Birth	Male	286 (80.1)	71 (19.9)	-	-	-	-	-
	Female	229 (83.6)	45 (16.4)	0.79 (0.52-1.19, p=0.266)	0.73 (0.47-1.11, p=0.144)	0.82 (0.51-1.31, p=0.414)	0.72 (0.46-1.10, p=0.133)	0.82 (0.51-1.31, p=0.405)
Age (years)	15-19	109 (85.2)	19 (14.8)	-	-	-	-	-
	< 1 m	34 (66.7)	17 (33.2)	2.87 (1.34-6.16, p=0.007)	2.76 (1.28-5.93, p=0.009)	2.95 (1.26-6.98, p=0.012)	3.02 (1.39-6.55, p=0.005)	3.21 (1.36-7.66, p=0.008)
	≥ 1 m <1 yr	152 (91.6)	14 (8.4)	0.53 (0.25-1.09, p=0.088)	0.50 (0.23-1.04, p=0.066)	0.48 (0.20-1.12, p=0.091)	0.56 (0.26-1.17, p=0.123)	0.53 (0.22-1.25, p=0.151)
	1-4	89 (85.6)	15 (14.4)	0.97 (0.46-2.01, p=0.928)	0.93 (0.44-1.94, p=0.856)	1.26 (0.56-2.83, p=0.574)	0.95 (0.45-1.98, p=0.889)	1.28 (0.57-2.89, p=0.545)
	5-9	73 (80.2)	18 (19.8)	1.41 (0.69-2.89, p=0.338)	1.36 (0.66-2.78, p=0.396)	1.31 (0.58-2.99, p=0.518)	1.36 (0.66-2.79, p=0.399)	1.33 (0.58-3.05, p=0.493)
	10-14	58 (63.7)	33 (36.3)	3.26 (1.72-6.33, p<0.001)	3.24 (1.71-6.30, p<0.001)	3.21 (1.54-6.92, p=0.002)	3.22 (1.69-6.28, p<0.001)	3.23 (1.55-6.99, p=0.002)
Ethnicity	White	281 (87.3)	41 (12.7)	-	-	-	-	-
	Black	36 (65.5)	19 (34.5)	3.62 (1.88-6.86, p<0.001)	-	2.87 (1.43-5.67, p=0.003)	-	2.82 (1.41-5.57, p=0.003)
	South Asian	49 (79.0)	13 (21.0)	1.82 (0.88-3.56, p=0.091)	-	1.89 (0.89-3.83, p=0.083)	-	1.86 (0.87-3.77, p=0.094)
	Other	92 (78.0)	26 (22.0)	1.94 (1.11-3.32, p=0.017)	-	1.92 (1.08-3.37, p=0.024)	-	1.91 (1.07-3.34, p=0.025)
Any comorbidity	No/Unknown	305 (85.2)	53 (14.8)	-	-	-	-	-
	Yes	210 (76.9)	63 (23.1)	1.73 (1.15-2.60, p=0.008)	-	-	1.56 (1.02-2.40, p=0.041)	1.42 (0.89-2.28, p=0.141)

Supp Table E. *Comorbidities stratified by critical care admission. Prematurity defined as gestation < 37 weeks. Prematurity data was only collected for children under 1 year old. Analysis by Fisher's exact test*

		Standard ward admission	Critical care admission	p
Total N (%)		516 (81.6)	116 (18.4)	
Prematurity	No	135 (26.2)	15 (12.9)	0.001
	Yes	30 (5.8)	15 (12.9)	
	(Missing)	351 (68.0)	86 (74.1)	
Neurological	No	448 (86.8)	97 (83.6)	0.064
	Yes	47 (9.1)	18 (15.5)	
	(Missing)	21 (4.1)	1 (0.9)	
Neurodisability	No	454 (88.0)	99 (85.3)	0.160
	Yes	23 (4.5)	9 (7.8)	
	(Missing)	39 (7.6)	8 (6.9)	
Respiratory	No	470 (91.1)	103 (88.8)	0.019
	Yes	21 (4.1)	12 (10.3)	
	(Missing)	25 (4.8)	1 (0.9)	
Asthma	No	455 (88.2)	111 (95.7)	0.234
	Yes	40 (7.8)	5 (4.3)	
	(Missing)	21 (4.1)	0 (0.0)	
Cardiac	No	468 (90.7)	102 (87.9)	0.018
	Yes	25 (4.8)	13 (11.2)	
	(Missing)	23 (4.5)	1 (0.9)	
Gastrointestinal	No	483 (93.6)	108 (93.1)	0.058
	Yes	11 (2.1)	7 (6.0)	
	(Missing)	22 (4.3)	1 (0.9)	
Haematology / Oncology / Immunology	No	454 (88.0)	109 (94.0)	0.335
	Yes	42 (8.1)	6 (5.2)	
	(Missing)	20 (3.9)	1 (0.9)	
Obesity	No	477 (92.4)	108 (93.1)	0.028
	Yes	10 (1.9)	7 (6.0)	
	(Missing)	29 (5.6)	1 (0.9)	
Malnutrition	No	485 (94.0)	113 (97.4)	0.321
	Yes	4 (0.8)	2 (1.7)	
	(Missing)	27 (5.2)	1 (0.9)	
Genetic	No	461 (89.3)	110 (94.8)	0.097
	Yes	21 (4.1)	1 (0.9)	
	(Missing)	34 (6.6)	5 (4.3)	
Other endocrine	No	474 (91.9)	109 (94.0)	1.000
	Yes	8 (1.6)	2 (1.7)	
	(Missing)	34 (6.6)	5 (4.3)	
Diabetes	No	481 (93.2)	114 (98.3)	0.748
	Yes	13 (2.5)	2 (1.7)	
	(Missing)	22 (4.3)	0 (0.0)	
Renal	No	483 (93.6)	113 (97.4)	1.000
	Yes	12 (2.3)	3 (2.6)	
	(Missing)	21 (4.1)	0 (0.0)	

Metabolic	No	480 (93.0)	111 (95.7)	1.000
	Yes	2 (0.4)	0 (0.0)	
	(Missing)	34 (6.6)	5 (4.3)	
Rheumatology	No	490 (95.0)	115 (99.1)	0.572
	Yes	3 (0.6)	1 (0.9)	
	(Missing)	23 (4.5)	0 (0.0)	
Other	No	467 (90.5)	111 (95.7)	0.087
	Yes	15 (2.9)	0 (0.0)	
	(Missing)	34 (6.6)	5 (4.3)	
Immunosuppressant use prior to presentation	No	443 (85.9)	101 (87.1)	1.000
	Yes	43 (8.3)	10 (8.6)	
	(Missing)	30 (5.8)	5 (4.3)	

.

Supp Table F. *Presenting symptoms stratified by critical care admission. Analysis by Fisher's exact test.*

		Standard ward admission	Critical care admission	p
Total N (%)		516 (81.6)	116 (18.4)	
History of fever	No	146 (28.3)	38 (32.8)	0.428
	Yes	353 (68.4)	76 (65.5)	
	(Missing)	17 (3.3)	2 (1.7)	
Cough	No	292 (56.6)	72 (62.1)	0.331
	Yes	193 (37.4)	38 (32.8)	
	(Missing)	31 (6.0)	6 (5.2)	
Cough: with sputum production	No	383 (74.2)	86 (74.1)	0.055
	Yes	26 (5.0)	12 (10.3)	
	(Missing)	107 (20.7)	18 (15.5)	
Sore throat	No	313 (60.7)	79 (68.1)	0.292
	Yes	41 (7.9)	15 (12.9)	
	(Missing)	162 (31.4)	22 (19.0)	
Runny nose (Rhinorrhoea)	No	340 (65.9)	89 (76.7)	0.001
	Yes	72 (14.0)	4 (3.4)	
	(Missing)	104 (20.2)	23 (19.8)	
Ear pain	No	347 (67.2)	79 (68.1)	0.008
	Yes	3 (0.6)	5 (4.3)	
	(Missing)	166 (32.2)	32 (27.6)	
Wheezing	No	401 (77.7)	96 (82.8)	0.332
	Yes	41 (7.9)	6 (5.2)	
	(Missing)	74 (14.3)	14 (12.1)	
Chest pain	No	348 (67.4)	81 (69.8)	0.207
	Yes	19 (3.7)	8 (6.9)	
	(Missing)	149 (28.9)	27 (23.3)	
Muscle aches (Myalgia)	No	314 (60.9)	70 (60.3)	0.047
	Yes	31 (6.0)	14 (12.1)	
	(Missing)	171 (33.1)	32 (27.6)	
Joint pain (Arthralgia)	No	333 (64.5)	81 (69.8)	0.746
	Yes	13 (2.5)	2 (1.7)	
	(Missing)	170 (32.9)	33 (28.4)	
Fatigue / Malaise	No	279 (54.1)	56 (48.3)	0.095
	Yes	108 (20.9)	33 (28.4)	
	(Missing)	129 (25.0)	27 (23.3)	
Shortness of breath (Dyspnea)	No	331 (64.1)	63 (54.3)	0.010
	Yes	128 (24.8)	44 (37.9)	
	(Missing)	57 (11.0)	9 (7.8)	
Lower chest wall indrawing	No	384 (74.4)	84 (72.4)	0.289
	Yes	30 (5.8)	10 (8.6)	
	(Missing)	102 (19.8)	22 (19.0)	
Headache	No	299 (57.9)	67 (57.8)	0.085
	Yes	44 (8.5)	17 (14.7)	
	(Missing)	173 (33.5)	32 (27.6)	
Altered consciousness / confusion	No	410 (79.5)	80 (69.0)	<0.001

	Yes	24 (4.7)	19 (16.4)	
	(Missing)	82 (15.9)	17 (14.7)	
Seizures	No	424 (82.2)	95 (81.9)	0.825
	Yes	29 (5.6)	7 (6.0)	
	(Missing)	63 (12.2)	14 (12.1)	
Abdominal pain	No	298 (57.8)	61 (52.6)	0.002
	Yes	74 (14.3)	33 (28.4)	
	(Missing)	144 (27.9)	22 (19.0)	
Nausea / Vomiting	No	324 (62.8)	58 (50.0)	0.004
	Yes	133 (25.8)	46 (39.7)	
	(Missing)	59 (11.4)	12 (10.3)	
Diarrhoea	No	390 (75.6)	64 (55.2)	<0.001
	Yes	58 (11.2)	40 (34.5)	
	(Missing)	68 (13.2)	12 (10.3)	
Conjunctivitis	No	402 (77.9)	84 (72.4)	<0.001
	Yes	10 (1.9)	16 (13.8)	
	(Missing)	104 (20.2)	16 (13.8)	
Skin rash	No	386 (74.8)	82 (70.7)	0.072
	Yes	64 (12.4)	23 (19.8)	
	(Missing)	66 (12.8)	11 (9.5)	
Lymphadenopathy	No	396 (76.7)	82 (70.7)	0.022
	Yes	14 (2.7)	9 (7.8)	
	(Missing)	106 (20.5)	25 (21.6)	
Bleeding	No	423 (82.0)	100 (86.2)	1.000
	Yes	8 (1.6)	2 (1.7)	
	(Missing)	85 (16.5)	14 (12.1)	

Supp Table G. *Clinical observations and Paediatric Early Waring Score (PEWS) at presentation stratified by critical care admission. RR = respiratory rate, SpO_2 = oxygen saturations, HR = heart rate, BP = blood pressure. 13 children did not have observations documented at presentation and as such PEWS could not be calculated. Total PEWS score analysed by Kruskal Wallis test, individual PEWS components analysed by Fisher's exact test.*

PEWS component		Standard ward admission	Critical care admission	p
Total N (%)		516 (81.6)	116 (18.4)	
Total PEWS	Median (IQR)	2.0 (1.0 to 4.0)	5.0 (2.0 to 7.0)	<0.001
PEWS RR	0	339 (65.7)	63 (54.3)	0.003
	1	115 (22.3)	32 (27.6)	
	3	26 (5.0)	15 (12.9)	
	(Missing)	36 (7.0)	6 (5.2)	
PEWS SpO_2	0	448 (86.8)	93 (80.2)	0.006
	1	15 (2.9)	8 (6.9)	
	3	15 (2.9)	9 (7.8)	
	(Missing)	38 (7.4)	6 (5.2)	
PEWS O_2 requirement	0	439 (85.1)	63 (54.3)	<0.001
	1	34 (6.6)	46 (39.7)	
	(Missing)	43 (8.3)	7 (6.0)	
PEWS HR	0	264 (51.2)	49 (42.2)	0.161
	1	101 (19.6)	30 (25.9)	
	3	118 (22.9)	29 (25.0)	
	(Missing)	33 (6.4)	8 (6.9)	
PEWS Systolic BP	0	129 (25.0)	35 (30.2)	0.144
	1	125 (24.2)	40 (34.5)	
	3	48 (9.3)	24 (20.7)	
	(Missing)	214 (41.5)	17 (14.7)	
PEWS Conscious level	0	436 (84.5)	60 (51.7)	<0.001
	3	16 (3.1)	39 (33.6)	
	(Missing)	64 (12.4)	17 (14.7)	
PEWS Capillary refill time	0	139 (26.9)	44 (37.9)	0.496
	1	83 (16.1)	32 (27.6)	
	(Missing)	294 (57.0)	40 (34.5)	
PEWS Temperature	0	315 (61.0)	69 (59.5)	0.197
	1	166 (32.2)	41 (35.3)	
	3	2 (0.4)	2 (1.7)	
	(Missing)	33 (6.4)	4 (3.4)	

Supp Table H. *Blood results at presentation stratified by critical care admission. WBC = white blood cell, APTT = activated partial thromboplastin time, PT = prothrombin time, INR -= international normalised ration, ALT = alanine transaminase, AST = aspartate aminotransferase, CRP = C-reactive protein. Analysis by Kruskal Wallis test.*

Investigation	Total n		Standard ward admission	Critical care admission	p
Total N (%)			516 (81.6)	116 (18.4)	
Haemoglobin (g/L)	455	Median (IQR)	119.0 (105.0 to 134.0)	111.0 (101.5 to 128.0)	0.044
Haematocrit (%)	179	Median (IQR)	34.6 (30.6 to 39.1)	34.0 (31.2 to 37.8)	0.953
WBC ($x10^9$/L)	453	Median (IQR)	9.1 (6.0 to 13.4)	10.5 (7.8 to 16.4)	0.005
Lymphocyte count ($x10^9$/L)	450	Median (IQR)	2.2 (1.3 to 3.8)	1.3 (0.8 to 2.4)	<0.001
Neutrophil count ($x10^9$/L)	451	Median (IQR)	4.6 (2.3 to 8.2)	7.7 (4.3 to 12.3)	<0.001
Platelet count ($x10^9$/L)	449	Median (IQR)	296.5 (229.0 to 383.5)	192.0 (133.0 to 280.5)	<0.001
PT (sec)	122	Median (IQR)	13.1 (12.0 to 14.4)	13.0 (11.9 to 15.0)	0.721
INR	42	Median (IQR)	1.1 (1.0 to 1.3)	1.2 (1.1 to 1.3)	0.177
ALT (IU/L)	302	Median (IQR)	23.0 (15.0 to 33.0)	31.5 (21.0 to 49.2)	<0.001
AST (IU/L)	66	Median (IQR)	35.0 (25.0 to 49.0)	57.0 (40.0 to 123.0)	0.024
Bilirubin (µmol/L)	345	Median (IQR)	7.0 (5.0 to 14.0)	8.0 (5.0 to 16.0)	0.804
Glucose (mmol/L)	181	Median (IQR)	5.3 (4.7 to 6.3)	6.2 (5.3 to 8.6)	<0.001
Urea (mmol/L)	402	Median (IQR)	3.5 (2.6 to 4.4)	4.2 (2.8 to 7.5)	0.004
Creatinine (µmol/L)	448	Median (IQR)	31.0 (21.0 to 52.8)	44.0 (27.0 to 70.8)	<0.001
Sodium (mmol/L)	465	Median (IQR)	137.0 (136.0 to 139.0)	137.0 (134.0 to 140.0)	0.102
Potassium (mmol/L)	451	Median (IQR)	4.4 (4.0 to 5.0)	4.0 (3.5 to 4.4)	<0.001
Lactate (mmol/L)	143	Median (IQR)	1.9 (1.3 to 2.7)	1.3 (1.0 to 2.4)	0.018
CRP (mg/L)	408	Median (IQR)	11.0 (3.0 to 54.5)	64.5 (11.1 to 200.2)	<0.001
Infiltrates on any CXR	243	No	110 (67.9)	34 (42.0)	<0.001
		Yes	52 (32.1)	47 (58.0)	

Supp Table I. Demographics stratified by admission to critical care, excluding patients with MIS-C. Categorical variables analysed using Fisher's exact test, continuous variables by Kruskal Wallis test.

Variable		Standard ward admission	Critical care admission	p
Total N (%)		502 (86.6)	78 (13.4)	
Age (years)	Median (IQR)	3.5 (0.3 to 13.8)	1.8 (0.1 to 12.5)	0.142
Neonate (< 1 month)	No	468 (93.2)	61 (78.2)	<0.001
	Yes	34 (6.8)	17 (21.8)	
Age (years)	<1	185 (36.9)	31 (39.7)	0.166
	1-4	88 (17.5)	13 (16.7)	
	5-9	69 (13.7)	6 (7.7)	
	10-14	54 (10.8)	15 (19.2)	
	15-19	106 (21.1)	13 (16.7)	
Sex at Birth	Male	277 (55.2)	49 (62.8)	0.222
	Female	224 (44.6)	29 (37.2)	
	(Missing)	1 (0.2)	0 (0.0)	
Ethnicity	White	275 (54.8)	32 (41.0)	0.028
	Black	34 (6.8)	12 (15.4)	
	South Asian	48 (9.6)	10 (12.8)	
	Other	89 (17.7)	13 (16.7)	
	(Missing)	56 (11.2)	11 (14.1)	
Admitted > 5 days before symptom onset	No	423 (84.3)	54 (69.2)	<0.001
	Yes	36 (7.2)	18 (23.1)	
	(Missing)	43 (8.6)	6 (7.7)	
Any comorbidity	No/Unknown	297 (59.2)	25 (32.1)	<0.001
	Yes	205 (40.8)	53 (67.9)	
Prematurity	No	134 (26.7)	15 (19.2)	0.001
	Yes	30 (6.0)	15 (19.2)	
	(Missing)	338 (67.3)	48 (61.5)	
Respiratory	No	457 (91.0)	67 (85.9)	0.004
	Yes	20 (4.0)	10 (12.8)	
	(Missing)	25 (5.0)	1 (1.3)	
Cardiac	No	455 (90.6)	65 (83.3)	0.002
	Yes	24 (4.8)	12 (15.4)	
	(Missing)	23 (4.6)	1 (1.3)	
Obesity	No	466 (92.8)	73 (93.6)	0.072
	Yes	8 (1.6)	4 (5.1)	
	(Missing)	28 (5.6)	1 (1.3)	
Immunosuppressant use prior to presentation	No	429 (85.5)	67 (85.9)	0.831
	Yes	43 (8.6)	7 (9.0)	
	(Missing)	30 (6.0)	4 (5.1)	

Supp Table J. *Comorbidities stratified by admission to critical care, excluding patients with MIS-C. Prematurity defined as gestation < 37 weeks. Prematurity data was only collected for children under 1 year old. Analysis by Fisher's exact test*

		Standard ward admission	Critical care admission	p
Total N (%)		502 (86.6)	78 (13.4)	
Prematurity	No	134 (26.7)	15 (19.2)	0.001
	Yes	30 (6.0)	15 (19.2)	
	(Missing)	338 (67.3)	48 (61.5)	
Neurological	No	435 (86.7)	60 (76.9)	0.003
	Yes	46 (9.2)	17 (21.8)	
	(Missing)	21 (4.2)	1 (1.3)	
Neurodisability	No	441 (87.8)	62 (79.5)	0.014
	Yes	22 (4.4)	9 (11.5)	
	(Missing)	39 (7.8)	7 (9.0)	
Respiratory	No	457 (91.0)	67 (85.9)	0.004
	Yes	20 (4.0)	10 (12.8)	
	(Missing)	25 (5.0)	1 (1.3)	
Asthma	No	442 (88.0)	76 (97.4)	0.100
	Yes	39 (7.8)	2 (2.6)	
	(Missing)	21 (4.2)	0 (0.0)	
Cardiac	No	455 (90.6)	65 (83.3)	0.002
	Yes	24 (4.8)	12 (15.4)	
	(Missing)	23 (4.6)	1 (1.3)	
Gastrointestinal	No	469 (93.4)	70 (89.7)	0.007
	Yes	11 (2.2)	7 (9.0)	
	(Missing)	22 (4.4)	1 (1.3)	
Haematology / Oncology / Immunology	No	440 (87.6)	71 (91.0)	1.000
	Yes	42 (8.4)	6 (7.7)	
	(Missing)	20 (4.0)	1 (1.3)	
Obesity	No	466 (92.8)	73 (93.6)	0.072
	Yes	8 (1.6)	4 (5.1)	
	(Missing)	28 (5.6)	1 (1.3)	
Malnutrition	No	472 (94.0)	76 (97.4)	0.529
	Yes	4 (0.8)	1 (1.3)	
	(Missing)	26 (5.2)	1 (1.3)	
Genetic	No	449 (89.4)	73 (93.6)	0.337
	Yes	20 (4.0)	1 (1.3)	
	(Missing)	33 (6.6)	4 (5.1)	
Other endocrine	No	462 (92.0)	72 (92.3)	0.353
	Yes	7 (1.4)	2 (2.6)	
	(Missing)	33 (6.6)	4 (5.1)	
Diabetes	No	468 (93.2)	76 (97.4)	1.000
	Yes	13 (2.6)	2 (2.6)	
	(Missing)	21 (4.2)	0 (0.0)	
Renal	No	469 (93.4)	76 (97.4)	1.000
	Yes	12 (2.4)	2 (2.6)	
	(Missing)	21 (4.2)	0 (0.0)	

Metabolic	No	467 (93.0)	74 (94.9)	1.000
	Yes	2 (0.4)	0 (0.0)	
	(Missing)	33 (6.6)	4 (5.1)	
Rheumatology	No	477 (95.0)	77 (98.7)	0.453
	Yes	3 (0.6)	1 (1.3)	
	(Missing)	22 (4.4)	0 (0.0)	
Other	No	454 (90.4)	74 (94.9)	0.242
	Yes	15 (3.0)	0 (0.0)	
	(Missing)	33 (6.6)	4 (5.1)	
Immunosuppressant use prior to presentation	No	429 (85.5)	67 (85.9)	0.831
	Yes	43 (8.6)	7 (9.0)	
	(Missing)	30 (6.0)	4 (5.1)	

__Supp Table K.__ Presenting symptoms stratified by critical care admission excluding patients with MIS-C. Analysis by Fisher's exact test.

		Standard ward admission	Critical care admission	P
Total N (%)		502 (86.6)	78 (13.4)	
History of fever	No	146 (29.1)	38 (48.7)	0.001
	Yes	339 (67.5)	38 (48.7)	
	(Missing)	17 (3.4)	2 (2.6)	
Cough	No	284 (56.6)	47 (60.3)	0.440
	Yes	187 (37.3)	25 (32.1)	
	(Missing)	31 (6.2)	6 (7.7)	
Cough: with sputum production	No	373 (74.3)	57 (73.1)	0.062
	Yes	23 (4.6)	8 (10.3)	
	(Missing)	106 (21.1)	13 (16.7)	
Sore throat	No	305 (60.8)	54 (69.2)	0.818
	Yes	37 (7.4)	5 (6.4)	
	(Missing)	160 (31.9)	19 (24.4)	
Runny nose (Rhinorrhoea)	No	328 (65.3)	56 (71.8)	0.037
	Yes	71 (14.1)	4 (5.1)	
	(Missing)	103 (20.5)	18 (23.1)	
Ear pain	No	334 (66.5)	53 (67.9)	0.146
	Yes	3 (0.6)	2 (2.6)	
	(Missing)	165 (32.9)	23 (29.5)	
Wheezing	No	389 (77.5)	61 (78.2)	1.000
	Yes	39 (7.8)	6 (7.7)	
	(Missing)	74 (14.7)	11 (14.1)	
Chest pain	No	335 (66.7)	52 (66.7)	1.000
	Yes	19 (3.8)	3 (3.8)	
	(Missing)	148 (29.5)	23 (29.5)	
Muscle aches (Myalgia)	No	305 (60.8)	50 (64.1)	0.402
	Yes	28 (5.6)	2 (2.6)	
	(Missing)	169 (33.7)	26 (33.3)	
Joint pain (Arthralgia)	No	321 (63.9)	52 (66.7)	0.230
	Yes	13 (2.6)	0 (0.0)	
	(Missing)	168 (33.5)	26 (33.3)	
Fatigue / Malaise	No	271 (54.0)	41 (52.6)	1.000
	Yes	102 (20.3)	15 (19.2)	
	(Missing)	129 (25.7)	22 (28.2)	
Shortness of breath (Dyspnea)	No	322 (64.1)	40 (51.3)	0.008
	Yes	123 (24.5)	31 (39.7)	
	(Missing)	57 (11.4)	7 (9.0)	
Lower chest wall indrawing	No	370 (73.7)	52 (66.7)	0.133
	Yes	30 (6.0)	8 (10.3)	
	(Missing)	102 (20.3)	18 (23.1)	
Headache	No	290 (57.8)	45 (57.7)	0.816
	Yes	40 (8.0)	5 (6.4)	
	(Missing)	172 (34.3)	28 (35.9)	

Altered consciousness / confusion	No	397 (79.1)	51 (65.4)	<0.001
	Yes	24 (4.8)	15 (19.2)	
	(Missing)	81 (16.1)	12 (15.4)	
Seizures	No	410 (81.7)	60 (76.9)	0.303
	Yes	29 (5.8)	7 (9.0)	
	(Missing)	63 (12.5)	11 (14.1)	
Abdominal pain	No	292 (58.2)	51 (65.4)	0.188
	Yes	67 (13.3)	6 (7.7)	
	(Missing)	143 (28.5)	21 (26.9)	
Nausea / Vomiting	No	320 (63.7)	50 (64.1)	0.885
	Yes	123 (24.5)	18 (23.1)	
	(Missing)	59 (11.8)	10 (12.8)	
Diarrhoea	No	383 (76.3)	54 (69.2)	0.055
	Yes	52 (10.4)	14 (17.9)	
	(Missing)	67 (13.3)	10 (12.8)	
Conjunctivitis	No	393 (78.3)	65 (83.3)	1.000
	Yes	5 (1.0)	1 (1.3)	
	(Missing)	104 (20.7)	12 (15.4)	
Skin rash	No	382 (76.1)	64 (82.1)	0.312
	Yes	54 (10.8)	5 (6.4)	
	(Missing)	66 (13.1)	9 (11.5)	
Lymphadenopathy	No	386 (76.9)	55 (70.5)	0.094
	Yes	10 (2.0)	4 (5.1)	
	(Missing)	106 (21.1)	19 (24.4)	
Bleeding	No	409 (81.5)	64 (82.1)	0.633
	Yes	8 (1.6)	2 (2.6)	
	(Missing)	85 (16.9)	12 (15.4)	

Supp Table L. *Clinical observations and Paediatric Early Waring Score (PEWS) at presentation stratified by critical care admission excluding patients with MIS-C. RR = respiratory rate, SpO_2 = oxygen saturations, HR = heart rate, BP = blood pressure. 12 children did not have observations on admission documented and as such PEWS could not be calculated. Total PEWS score analysed by Kruskal Wallis test, individual PEWS components analysed by Fisher's exact test.*

		Standard ward admission	Critical care admission	P
Total N (%)		502 (86.6)	78 (13.4)	
Total PEWS	Median (IQR)	2.0 (1.0 to 4.0)	4.5 (2.0 to 7.0)	<0.001
PEWS RR	0	332 (66.1)	42 (53.8)	0.032
	1	110 (21.9)	23 (29.5)	
	3	24 (4.8)	8 (10.3)	
	(Missing)	36 (7.2)	5 (6.4)	
PEWS SpO_2	0	435 (86.7)	58 (74.4)	<0.001
	1	15 (3.0)	8 (10.3)	
	3	14 (2.8)	8 (10.3)	
	(Missing)	38 (7.6)	4 (5.1)	
PEWS O_2 requirement	0	429 (85.5)	37 (47.4)	<0.001
	1	31 (6.2)	36 (46.2)	
	(Missing)	42 (8.4)	5 (6.4)	
PEWS HR	0	259 (51.6)	38 (48.7)	0.886
	1	95 (18.9)	16 (20.5)	
	3	115 (22.9)	18 (23.1)	
	(Missing)	33 (6.6)	6 (7.7)	
PEWS Systolic BP	0	125 (24.9)	21 (26.9)	0.257
	1	116 (23.1)	26 (33.3)	
	3	48 (9.6)	15 (19.2)	
	(Missing)	213 (42.4)	16 (20.5)	
PEWS Conscious level	0	422 (84.1)	36 (46.2)	<0.001
	3	16 (3.2)	26 (33.3)	
	(Missing)	64 (12.7)	16 (20.5)	
PEWS Capillary refill time	0	137 (27.3)	25 (32.1)	0.140
	1	77 (15.3)	23 (29.5)	
	(Missing)	288 (57.4)	30 (38.5)	
PEWS Temperature	0	311 (62.0)	52 (66.7)	0.077
	1	156 (31.1)	20 (25.6)	
	3	2 (0.4)	2 (2.6)	
	(Missing)	33 (6.6)	4 (5.1)	

Supp Table M. *Blood results at presentation stratified by critical care admission excluding patients with MIS-C. WBC = white blood cell, APTT = activated partial thromboplastin time, PT = prothrombin time, INR -= international normalised ration, ALT = alanine transaminase, AST = aspartate aminotransferase, CRP = C-reactive protein. Analysis by Kruskal Wallis test.*

Investigation	Total n		Standard ward admission	Critical care Admission	p
Total N (%)			502 (86.6)	78 (13.4)	
Haemoglobin (g/L)	405	Median (IQR)	119.0 (105.0 to 134.0)	114.0 (101.0 to 139.8)	0.500
Haematocrit (%)	162	Median (IQR)	33.9 (30.6 to 39.0)	35.0 (31.2 to 39.7)	0.608
WBC (x10^9/L)	403	Median (IQR)	9.1 (6.0 to 13.5)	9.8 (7.6 to 16.2)	0.094
Lymphocyte count (x10^9/L)	400	Median (IQR)	2.2 (1.3 to 3.8)	2.0 (1.1 to 3.6)	0.297
Neutrophil count (x10^9/L)	401	Median (IQR)	4.5 (2.3 to 8.2)	6.2 (3.4 to 9.6)	0.059
Platelet count (x10^9/L)	399	Median (IQR)	297.0 (229.0 to 387.0)	236.5 (132.5 to 346.0)	0.001
PT (sec)	93	Median (IQR)	12.9 (12.0 to 14.2)	13.5 (12.4 to 16.4)	0.110
INR	31	Median (IQR)	1.1 (1.0 to 1.3)	1.1 (1.1 to 1.3)	0.287
ALT (IU/L)	257	Median (IQR)	23.0 (15.0 to 32.8)	29.0 (18.5 to 41.0)	0.146
AST (IU/L)	53	Median (IQR)	36.0 (25.5 to 54.0)	47.5 (31.0 to 129.8)	0.237
Bilirubin (μmol/L)	301	Median (IQR)	7.0 (5.0 to 14.0)	7.0 (5.0 to 15.2)	0.888
Glucose (mmol/L)	152	Median (IQR)	5.3 (4.6 to 6.5)	6.4 (5.3 to 8.8)	0.005
Urea (mmol/L)	357	Median (IQR)	3.5 (2.6 to 4.4)	4.2 (2.6 to 5.4)	0.087
Creatinine (μmol/L)	400	Median (IQR)	30.0 (20.0 to 53.0)	32.0 (20.5 to 63.0)	0.585
Sodium (mmol/L)	414	Median (IQR)	137.0 (136.0 to 139.0)	138.0 (136.0 to 141.0)	0.232
Potassium (mmol/L)	400	Median (IQR)	4.4 (4.0 to 5.0)	4.3 (3.7 to 4.8)	0.063
Lactate (mmol/L)	119	Median (IQR)	1.9 (1.2 to 2.7)	1.4 (0.9 to 2.7)	0.128
CRP (mg/L)	364	Median (IQR)	9.0 (3.0 to 46.9)	21.4 (5.0 to 51.9)	0.069
Infiltrates on any CXR	203	No	107 (69.9)	21 (42.0)	0.001
		Yes	46 (30.1)	29 (58.0)	

***Supp Table N**. Comorbidities stratified by Multisystem Inflammatory Disease in Children and Adolescents (MIS-C). Immunosuppressant use includes oral but not inhaled steroids. Prematurity = born < 37 weeks gestation. Analysis by Fisher's exact test.*

Comorbidity		Did not meet MIS-C criteria	Met MIS-C criteria	p
Total N (%)		404 (88.6)	52 (11.4)	
Prematurity	No	113 (28.0)	1 (1.9)	1.000
	Yes	33 (8.2)	0 (0.0)	
	(Missing)	258 (63.9)	51 (98.1)	
Neurological	No	352 (87.1)	50 (96.2)	0.205
	Yes	40 (9.9)	2 (3.8)	
	(Missing)	12 (3.0)	0 (0.0)	
Neurodisability	No	358 (88.6)	50 (96.2)	0.492
	Yes	20 (5.0)	1 (1.9)	
	(Missing)	26 (6.4)	1 (1.9)	
Respiratory	No	367 (90.8)	49 (94.2)	1.000
	Yes	22 (5.4)	3 (5.8)	
	(Missing)	15 (3.7)	0 (0.0)	
Asthma	No	369 (91.3)	48 (92.3)	0.555
	Yes	24 (5.9)	4 (7.7)	
	(Missing)	11 (2.7)	0 (0.0)	
Cardiac	No	362 (89.6)	50 (96.2)	0.557
	Yes	27 (6.7)	2 (3.8)	
	(Missing)	15 (3.7)	0 (0.0)	
Gastrointestinal	No	375 (92.8)	52 (100.0)	0.241
	Yes	17 (4.2)	0 (0.0)	
	(Missing)	12 (3.0)	0 (0.0)	
Haematology / Oncology / Immunology	No	357 (88.4)	52 (100.0)	0.024
	Yes	35 (8.7)	0 (0.0)	
	(Missing)	12 (3.0)	0 (0.0)	
Obesity	No	379 (93.8)	46 (88.5)	0.005
	Yes	6 (1.5)	5 (9.6)	
	(Missing)	19 (4.7)	1 (1.9)	
Malnutrition	No	383 (94.8)	50 (96.2)	0.392
	Yes	3 (0.7)	1 (1.9)	
	(Missing)	18 (4.5)	1 (1.9)	
Genetic	No	366 (90.6)	49 (94.2)	1.000
	Yes	12 (3.0)	1 (1.9)	
	(Missing)	26 (6.4)	2 (3.8)	
Other endocrine	No	374 (92.6)	49 (94.2)	0.464
	Yes	4 (1.0)	1 (1.9)	
	(Missing)	26 (6.4)	2 (3.8)	
Diabetes	No	380 (94.1)	51 (98.1)	0.376
	Yes	12 (3.0)	0 (0.0)	
	(Missing)	12 (3.0)	1 (1.9)	
Renal	No	380 (94.1)	51 (98.1)	1.000
	Yes	12 (3.0)	1 (1.9)	
	(Missing)	12 (3.0)	0 (0.0)	

Metabolic	No	377 (93.3)	50 (96.2)	1.000
	Yes	1 (0.2)	0 (0.0)	
	(Missing)	26 (6.4)	2 (3.8)	
Rheumatology	No	389 (96.3)	51 (98.1)	1.000
	Yes	2 (0.5)	0 (0.0)	
	(Missing)	13 (3.2)	1 (1.9)	
Other	No	366 (90.6)	50 (96.2)	0.375
	Yes	12 (3.0)	0 (0.0)	
	(Missing)	26 (6.4)	2 (3.8)	
Immunosuppressant use prior to presentation	No	348 (86.1)	48 (92.3)	0.601
	Yes	35 (8.7)	3 (5.8)	
	(Missing)	21 (5.2)	1 (1.9)	

Supp Table O. *Presenting symptoms stratified by Multisystem Inflammatory Syndrome in Children and Adolescents (MIS-C). Analysis by Fisher's exact test.*

Symptom		Did not meet MIS-C criteria	Met MIS_C criteria	p
Total N (%)		404 (88.6)	52 (11.4)	
History of fever	No	106 (26.2)	0 (0.0)	<0.001
	Yes	296 (73.3)	52 (100.0)	
	(Missing)	2 (0.5)	0 (0.0)	
Cough	No	235 (58.2)	33 (63.5)	0.879
	Yes	148 (36.6)	19 (36.5)	
	(Missing)	21 (5.2)	0 (0.0)	
Cough: with sputum production	No	305 (75.5)	39 (75.0)	0.061
	Yes	20 (5.0)	7 (13.5)	
	(Missing)	79 (19.6)	6 (11.5)	
Sore throat	No	250 (61.9)	33 (63.5)	0.003
	Yes	34 (8.4)	14 (26.9)	
	(Missing)	120 (29.7)	5 (9.6)	
Runny nose (Rhinorrhoea)	No	277 (68.6)	45 (86.5)	0.011
	Yes	50 (12.4)	1 (1.9)	
	(Missing)	77 (19.1)	6 (11.5)	
Ear pain	No	273 (67.6)	39 (75.0)	0.051
	Yes	4 (1.0)	3 (5.8)	
	(Missing)	127 (31.4)	10 (19.2)	
Wheezing	No	321 (79.5)	47 (90.4)	0.753
	Yes	22 (5.4)	2 (3.8)	
	(Missing)	61 (15.1)	3 (5.8)	
Chest pain	No	270 (66.8)	42 (80.8)	0.195
	Yes	16 (4.0)	5 (9.6)	
	(Missing)	118 (29.2)	5 (9.6)	
Muscle aches (Myalgia)	No	249 (61.6)	29 (55.8)	<0.001
	Yes	21 (5.2)	15 (28.8)	
	(Missing)	134 (33.2)	8 (15.4)	
Joint pain (Arthralgia)	No	264 (65.3)	41 (78.8)	0.650
	Yes	9 (2.2)	2 (3.8)	
	(Missing)	131 (32.4)	9 (17.3)	
Fatigue / Malaise	No	216 (53.5)	23 (44.2)	0.004
	Yes	86 (21.3)	24 (46.2)	
	(Missing)	102 (25.2)	5 (9.6)	
Shortness of breath (Dyspnea)	No	250 (61.9)	32 (61.5)	0.421
	Yes	110 (27.2)	18 (34.6)	
	(Missing)	44 (10.9)	2 (3.8)	
Lower chest wall indrawing	No	293 (72.5)	46 (88.5)	0.400
	Yes	28 (6.9)	2 (3.8)	
	(Missing)	83 (20.5)	4 (7.7)	
Headache	No	237 (58.7)	31 (59.6)	<0.001
	Yes	26 (6.4)	16 (30.8)	

	(Missing)	141 (34.9)	5 (9.6)	
Altered consciousness / confusion	No	312 (77.2)	42 (80.8)	0.783
	Yes	28 (6.9)	4 (7.7)	
	(Missing)	64 (15.8)	6 (11.5)	
Seizures	No	335 (82.9)	49 (94.2)	0.092
	Yes	22 (5.4)	0 (0.0)	
	(Missing)	47 (11.6)	3 (5.8)	
Abdominal pain	No	236 (58.4)	16 (30.8)	<0.001
	Yes	56 (13.9)	34 (65.4)	
	(Missing)	112 (27.7)	2 (3.8)	
Nausea / Vomiting	No	252 (62.4)	12 (23.1)	<0.001
	Yes	112 (27.7)	38 (73.1)	
	(Missing)	40 (9.9)	2 (3.8)	
Diarrhoea	No	308 (76.2)	17 (32.7)	<0.001
	Yes	50 (12.4)	32 (61.5)	
	(Missing)	46 (11.4)	3 (5.8)	
Conjunctivitis	No	322 (79.7)	28 (53.8)	<0.001
	Yes	4 (1.0)	20 (38.5)	
	(Missing)	78 (19.3)	4 (7.7)	
Skin rash	No	314 (77.7)	22 (42.3)	<0.001
	Yes	45 (11.1)	28 (53.8)	
	(Missing)	45 (11.1)	2 (3.8)	
Lymphadenopathy	No	308 (76.2)	37 (71.2)	<0.001
	Yes	10 (2.5)	9 (17.3)	
	(Missing)	86 (21.3)	6 (11.5)	
Bleeding	No	332 (82.2)	50 (96.2)	0.611
	Yes	9 (2.2)	0 (0.0)	
	(Missing)	63 (15.6)	2 (3.8)	

Supp Table P. *Clinical observations and Paediatric Early Waring Score (PEWS) at presentation stratified by Multisystem Inflammatory Syndrome in Children and Adolescents (MIS-C). RR = respiratory rate, SpO_2 = oxygen saturations, HR = heart rate, BP = blood pressure. Total PEWS score analysed by Kruskal Wallis test. Individual PEWS components analysed by Fisher's exact test.*

PEWS component		Did not meet MIS-C criteria	Met MIS-C criteria	p
Total N (%)		404 (88.6)	52 (11.4)	
Total PEWS	Median (IQR)	3.0 (1.0 to 5.0)	5.0 (2.8 to 6.2)	<0.001
PEWS RR	0	257 (63.6)	28 (53.8)	0.032
	1	102 (25.2)	14 (26.9)	
	3	26 (6.4)	9 (17.3)	
	(Missing)	19 (4.7)	1 (1.9)	
PEWS SpO_2	0	354 (87.6)	48 (92.3)	0.374
	1	17 (4.2)	0 (0.0)	
	3	14 (3.5)	2 (3.8)	
	(Missing)	19 (4.7)	2 (3.8)	
PEWS O_2 requirement	0	328 (81.2)	36 (69.2)	0.030
	1	51 (12.6)	13 (25.0)	
	(Missing)	25 (6.2)	3 (5.8)	
PEWS HR	0	191 (47.3)	16 (30.8)	0.018
	1	87 (21.5)	20 (38.5)	
	3	109 (27.0)	14 (26.9)	
	(Missing)	17 (4.2)	2 (3.8)	
PEWS Systolic BP	0	100 (24.8)	18 (34.6)	0.699
	1	98 (24.3)	23 (44.2)	
	3	51 (12.6)	9 (17.3)	
	(Missing)	155 (38.4)	2 (3.8)	
PEWS Conscious level	0	317 (78.5)	38 (73.1)	0.003
	3	33 (8.2)	13 (25.0)	
	(Missing)	54 (13.4)	1 (1.9)	
PEWS Capillary refill time	0	110 (27.2)	21 (40.4)	1.000
	1	79 (19.6)	15 (28.8)	
	(Missing)	215 (53.2)	16 (30.8)	
PEWS Temperature	0	249 (61.6)	21 (40.4)	0.003
	1	139 (34.4)	31 (59.6)	
	3	2 (0.5)	0 (0.0)	
	(Missing)	14 (3.5)	0 (0.0)	

Supp Table Q. *Blood results at presentation stratified by Multisystem Inflammatory Syndrome in Children and Adolescents (MIS-C). WBC = white blood cell, APTT = activated partial thromboplastin time, PT = prothrombin time, INR = international normalised ration, ALT = alanine transaminase, AST = aspartate aminotransferase, CRP = C-reactive protein. Analysis by Kruskal Wallis test.*

Investigation	Total n		Did not meet MIS-C criteria	Met MIS-C criteria	p
Haemoglobin (g/L)	403	Median (IQR)	118.0 (104.0 to 134.0)	110.0 (104.0 to 120.0)	0.023
Haematocrit (%)	153	Median (IQR)	33.8 (30.0 to 39.0)	34.0 (31.8 to 39.0)	0.765
WBC ($x10^9$/L)	401	Median (IQR)	9.2 (6.4 to 13.8)	10.6 (8.1 to 15.3)	0.050
Lymphocyte count ($x10^9$/L)	400	Median (IQR)	2.2 (1.3 to 3.8)	0.9 (0.7 to 1.7)	<0.001
Neutrophil count ($x10^9$/L)	401	Median (IQR)	4.6 (2.3 to 8.6)	8.3 (5.9 to 12.4)	<0.001
Platelet count ($x10^9$/L)	398	Median (IQR)	293.0 (221.8 to 382.0)	177.0 (134.5 to 244.8)	<0.001
APTT (sec)	121	Median (IQR)	31.5 (28.0 to 35.6)	34.1 (29.4 to 38.4)	0.204
PT (sec)	109	Median (IQR)	13.1 (12.0 to 14.4)	13.0 (11.3 to 15.0)	0.773
INR	38	Median (IQR)	1.1 (1.0 to 1.3)	1.2 (1.1 to 1.2)	0.614
ALT (IU/L)	274	Median (IQR)	24.0 (15.0 to 35.0)	32.0 (23.0 to 50.0)	0.002
Bilirubin (μmol/L)	311	Median (IQR)	7.0 (4.5 to 14.0)	8.5 (5.0 to 16.2)	0.539
AST (IU/L)	61	Median (IQR)	39.0 (26.0 to 59.8)	63.0 (30.0 to 89.0)	0.252
Glucose (mmol/L)	163	Median (IQR)	5.5 (4.7 to 6.8)	6.1 (5.3 to 6.6)	0.093
Urea (mmol/L)	357	Median (IQR)	3.5 (2.6 to 4.4)	4.2 (3.2 to 9.2)	0.001
Lactate (mmol/L)	129	Median (IQR)	1.9 (1.1 to 2.7)	1.3 (1.1 to 2.0)	0.128
Creatinine (μmol/L)	398	Median (IQR)	30.0 (20.0 to 51.8)	55.0 (35.8 to 82.5)	<0.001
Sodium (mmol/L)	411	Median (IQR)	137.0 (136.0 to 139.0)	135.0 (133.0 to 137.0)	<0.001
Potassium (mmol/L)	400	Median (IQR)	4.4 (4.0 to 5.0)	3.8 (3.3 to 4.2)	<0.001
CRP (mg/L)	403	Median (IQR)	11.0 (4.0 to 49.0)	181.0 (99.8 to 266.2)	<0.001
Platelet count < 150 (x 10^9/L)	398	No	310 (89.1)	34 (68.0)	<0.001
		Yes	38 (10.9)	16 (32.0)	
Infiltrates on any CXR	215	No	110 (62.9)	16 (40.0)	0.012
		Yes	65 (37.1)	24 (60.0)	

Supp Table R. *Demographics at presentation and therapies administered in patients with Multisystem Inflammatory Syndrome in Children and Adolescents (MIS-C) stratified by detection method. Categorical variables analysed using Fisher's exact test, continuous variables by Kruskal Wallis test. IV = intravenous, IVIg = intravenous immunoglobulin.*

		PCR positive	Serology positive only	p
Total N (%)		28 (56.0)	22 (44.0)	
Age (years)	Median (IQR)	12.4 (8.9 to 15.3)	10.0 (7.7 to 13.2)	0.057
Age (years)	<1	0 (0.0)	1 (4.5)	0.009
	1-4	3 (10.7)	1 (4.5)	
	5-9	6 (21.4)	9 (40.9)	
	10-14	10 (35.7)	11 (50.0)	
	15-19	9 (32.1)	0 (0.0)	
Sex at Birth	Male	17 (60.7)	14 (63.6)	1.000
	Female	11 (39.3)	8 (36.4)	
Ethnicity	White	12 (42.9)	2 (9.1)	0.008
	Black	2 (7.1)	7 (31.8)	
	South Asian	2 (7.1)	2 (9.1)	
	Other	6 (21.4)	10 (45.5)	
	(Missing)	6 (21.4)	1 (4.5)	
White ethnicity	Non-White	10 (35.7)	19 (86.4)	0.003
	White	12 (42.9)	2 (9.1)	
	(Missing)	6 (21.4)	1 (4.5)	
Any comorbidity	No/Unknown	17 (60.7)	19 (86.4)	0.061
	Yes	11 (39.3)	3 (13.6)	
Obesity	No	22 (78.6)	22 (100.0)	0.056
	Yes	5 (17.9)	0 (0.0)	
	(Missing)	1 (3.6)	0 (0.0)	
Immunosuppressant use prior to presentation	No	26 (92.9)	20 (90.9)	0.581
	Yes	1 (3.6)	2 (9.1)	
	(Missing)	1 (3.6)	0 (0.0)	
Admitted > 5 days before symptom onset	No	28 (100.0)	22 (100.0)	1.000
	Yes	0 (0.0)	0 (0.0)	
Cardiac complication	No	11 (39.3)	5 (22.7)	0.022
	Yes	6 (21.4)	15 (68.2)	
	(Missing)	11 (39.3)	2 (9.1)	
IV steroid	No	15 (53.6)	3 (13.6)	0.002
	Yes	8 (28.6)	16 (72.7)	
	(Missing)	5 (17.9)	3 (13.6)	
IVIg	No	12 (42.9)	3 (13.6)	0.004
	Yes	9 (32.1)	19 (86.4)	
	(Missing)	7 (25.0)	0 (0.0)	
Immunomodulator	No	16 (57.1)	19 (86.4)	0.691
	Yes	4 (14.3)	3 (13.6)	
	(Missing)	8 (28.6)	0 (0.0)	
High flow nasal cannula oxygen	No	15 (53.6)	13 (59.1)	0.778
	Yes	13 (46.4)	9 (40.9)	
Non-invasive ventilation	No	18 (64.3)	15 (68.2)	1.000
	Yes	10 (35.7)	7 (31.8)	

Invasive ventilation	No	17 (60.7)	19 (86.4)	0.061
	Yes	11 (39.3)	3 (13.6)	
Inotropic support	No	13 (46.4)	10 (45.5)	0.772
	Yes	12 (42.9)	12 (54.5)	
	(Missing)	3 (10.7)	0 (0.0)	
ICU/HDU admission	No	9 (32.1)	4 (18.2)	0.339
	Yes	19 (67.9)	18 (81.8)	
Outcome	Discharged alive	20 (71.4)	21 (95.5)	0.059
	On-going care	7 (25.0)	1 (4.5)	
	(Missing)	1 (3.6)	0 (0.0)	

***Supp Table S**. Comorbidities in patients with Multisystem Inflammatory Syndrome in Children and Adolescents (MIS-C) stratified by detection method. Immunosuppressant use includes oral but not inhaled steroids. Prematurity = born < 37 weeks gestation. Analysis by Fisher's exact test.*

Comorbidity		PCR positive	Serology positive only	p
Total N (%)		28 (56.0)	22 (44.0)	
Neurological	No	26 (92.9)	22 (100.0)	0.497
	Yes	2 (7.1)	0 (0.0)	
Neurodisability	No	26 (92.9)	22 (100.0)	1.000
	Yes	1 (3.6)	0 (0.0)	
	(Missing)	1 (3.6)	0 (0.0)	
Respiratory	No	26 (92.9)	22 (100.0)	0.497
	Yes	2 (7.1)	0 (0.0)	
Asthma	No	26 (92.9)	20 (90.9)	1.000
	Yes	2 (7.1)	2 (9.1)	
Cardiac	No	27 (96.4)	22 (100.0)	1.000
	Yes	1 (3.6)	0 (0.0)	
Gastrointestinal	No	28 (100.0)	22 (100.0)	1.000
	Yes	0 (0.0)	0 (0.0)	
Haematology / Oncology / Immunology	No	28 (100.0)	22 (100.0)	1.000
	Yes	0 (0.0)	0 (0.0)	
Obesity	No	22 (78.6)	22 (100.0)	0.056
	Yes	5 (17.9)	0 (0.0)	
	(Missing)	1 (3.6)	0 (0.0)	
Malnutrition	No	26 (92.9)	22 (100.0)	1.000
	Yes	1 (3.6)	0 (0.0)	
	(Missing)	1 (3.6)	0 (0.0)	
Genetic	No	27 (96.4)	21 (95.5)	1.000
	Yes	0 (0.0)	0 (0.0)	
	(Missing)	1 (3.6)	1 (4.5)	
Other endocrine	No	26 (92.9)	21 (95.5)	1.000
	Yes	1 (3.6)	0 (0.0)	
	(Missing)	1 (3.6)	1 (4.5)	
Diabetes	No	27 (96.4)	22 (100.0)	1.000
	Yes	0 (0.0)	0 (0.0)	
	(Missing)	1 (3.6)	0 (0.0)	
Renal	No	28 (100.0)	21 (95.5)	0.440
	Yes	0 (0.0)	1 (4.5)	
Metabolic	No	27 (96.4)	21 (95.5)	1.000
	Yes	0 (0.0)	0 (0.0)	
	(Missing)	1 (3.6)	1 (4.5)	
Rheumatology	No	28 (100.0)	22 (100.0)	1.000
	Yes	0 (0.0)	0 (0.0)	
	(Missing)	0 (0.0)	0 (0.0)	
Other	No	27 (96.4)	21 (95.5)	1.000
	Yes	0 (0.0)	0 (0.0)	
	(Missing)	1 (3.6)	1 (4.5)	
Immunosuppressant use prior to presentation	No	26 (92.9)	20 (90.9)	0.581

Yes	1 (3.6)	2 (9.1)
(Missing)	1 (3.6)	0 (0.0)

***Supp Table T**. Presenting symptoms in patients with Multisystem Inflammatory Syndrome in Children and Adolescents (MIS-C) stratified by detection method. Analysis by Fisher's exact test.*

Symptoms		PCR positive	Serology positive only	p
Total N (%)		28 (56.0)	22 (44.0)	
History of fever	No	0 (0.0)	0 (0.0)	1.000
	Yes	28 (100.0)	22 (100.0)	
Cough	No	14 (50.0)	17 (77.3)	0.078
	Yes	14 (50.0)	5 (22.7)	
Cough: with sputum production	No	18 (64.3)	19 (86.4)	0.106
	Yes	6 (21.4)	1 (4.5)	
	(Missing)	4 (14.3)	2 (9.1)	
Sore throat	No	16 (57.1)	15 (68.2)	0.525
	Yes	9 (32.1)	5 (22.7)	
	(Missing)	3 (10.7)	2 (9.1)	
Runny nose (Rhinorrhoea)	No	24 (85.7)	20 (90.9)	1.000
	Yes	1 (3.6)	0 (0.0)	
	(Missing)	3 (10.7)	2 (9.1)	
Ear pain	No	22 (78.6)	15 (68.2)	1.000
	Yes	2 (7.1)	1 (4.5)	
	(Missing)	4 (14.3)	6 (27.3)	
Wheezing	No	25 (89.3)	20 (90.9)	0.500
	Yes	2 (7.1)	0 (0.0)	
	(Missing)	1 (3.6)	2 (9.1)	
Chest pain	No	24 (85.7)	16 (72.7)	1.000
	Yes	3 (10.7)	2 (9.1)	
	(Missing)	1 (3.6)	4 (18.2)	
Muscle aches (Myalgia)	No	16 (57.1)	11 (50.0)	0.754
	Yes	8 (28.6)	7 (31.8)	
	(Missing)	4 (14.3)	4 (18.2)	
Joint pain (Arthralgia)	No	22 (78.6)	17 (77.3)	1.000
	Yes	1 (3.6)	1 (4.5)	
	(Missing)	5 (17.9)	4 (18.2)	
Fatigue / Malaise	No	14 (50.0)	8 (36.4)	0.236
	Yes	10 (35.7)	13 (59.1)	
	(Missing)	4 (14.3)	1 (4.5)	
Shortness of breath (Dyspnea)	No	13 (46.4)	18 (81.8)	0.014
	Yes	14 (50.0)	3 (13.6)	
	(Missing)	1 (3.6)	1 (4.5)	
Lower chest wall indrawing	No	23 (82.1)	21 (95.5)	0.493
	Yes	2 (7.1)	0 (0.0)	
	(Missing)	3 (10.7)	1 (4.5)	
Headache	No	18 (64.3)	11 (50.0)	0.534
	Yes	8 (28.6)	8 (36.4)	
	(Missing)	2 (7.1)	3 (13.6)	
Altered consciousness / confusion	No	23 (82.1)	17 (77.3)	0.634

	Yes	3 (10.7)	1 (4.5)	
	(Missing)	2 (7.1)	4 (18.2)	
Seizures	No	27 (96.4)	20 (90.9)	1.000
	Yes	0 (0.0)	0 (0.0)	
	(Missing)	1 (3.6)	2 (9.1)	
Abdominal pain	No	15 (53.6)	1 (4.5)	<0.001
	Yes	12 (42.9)	20 (90.9)	
	(Missing)	1 (3.6)	1 (4.5)	
Nausea / Vomiting	No	6 (21.4)	5 (22.7)	1.000
	Yes	21 (75.0)	16 (72.7)	
	(Missing)	1 (3.6)	1 (4.5)	
Diarrhoea	No	11 (39.3)	5 (22.7)	0.355
	Yes	16 (57.1)	15 (68.2)	
	(Missing)	1 (3.6)	2 (9.1)	
Conjunctivitis	No	21 (75.0)	6 (27.3)	<0.001
	Yes	4 (14.3)	15 (68.2)	
	(Missing)	3 (10.7)	1 (4.5)	
Skin rash	No	13 (46.4)	8 (36.4)	0.565
	Yes	14 (50.0)	13 (59.1)	
	(Missing)	1 (3.6)	1 (4.5)	
Lymphadenopathy	No	22 (78.6)	13 (59.1)	0.451
	Yes	4 (14.3)	5 (22.7)	
	(Missing)	2 (7.1)	4 (18.2)	
Bleeding	No	27 (96.4)	21 (95.5)	1.000
	Yes	0 (0.0)	0 (0.0)	
	(Missing)	1 (3.6)	1 (4.5)	

Supp Table U. *Clinical observations and Paediatric Early Waring Score (PEWS) at presentation in patients with Multisystem Inflammatory Syndrome in Children and Adolescents (MIS-C) stratified by detection method. PEWS = Paediatric Early Warning Score. RR = respiratory rate, SpO_2 = oxygen saturations, HR = heart rate, BP = blood pressure. Total PEWS score analysed by Kruskal Wallis test. Individual PEWS components analysed by Fisher's exact test.*

PEWS component		PCR positive	Serology positive only	P
Total N (%)		28 (56.0)	22 (44.0)	
Total PEWS	Median (IQR)	5.0 (2.8 to 7.2)	4.5 (3.0 to 6.0)	0.340
PEWS RR	0	13 (46.4)	13 (59.1)	0.744
	1	8 (28.6)	6 (27.3)	
	3	6 (21.4)	3 (13.6)	
	(Missing)	1 (3.6)	0 (0.0)	
PEWS SpO_2	0	24 (85.7)	22 (100.0)	0.493
	3	2 (7.1)	0 (0.0)	
	(Missing)	2 (7.1)	0 (0.0)	
PEWS O_2 requirement	0	15 (53.6)	19 (86.4)	0.056
	1	10 (35.7)	3 (13.6)	
	(Missing)	3 (10.7)	0 (0.0)	
PEWS HR	0	9 (32.1)	7 (31.8)	0.876
	1	9 (32.1)	9 (40.9)	
	3	8 (28.6)	6 (27.3)	
	(Missing)	2 (7.1)	0 (0.0)	
PEWS Systolic BP	0	7 (25.0)	9 (40.9)	0.357
	1	15 (53.6)	8 (36.4)	
	3	4 (14.3)	5 (22.7)	
	(Missing)	2 (7.1)	0 (0.0)	
PEWS Conscious level	0	19 (67.9)	17 (77.3)	0.748
	3	8 (28.6)	5 (22.7)	
	(Missing)	1 (3.6)	0 (0.0)	
PEWS Capillary refill time	0	8 (28.6)	13 (59.1)	0.086
	1	10 (35.7)	4 (18.2)	
	(Missing)	10 (35.7)	5 (22.7)	
PEWS Temperature	0	11 (39.3)	10 (45.5)	0.775
	1	17 (60.7)	12 (54.5)	

Supp Table V. *Blood results at presentation in patients with Multisystem Inflammatory Syndrome in Children and Adolescents (MIS-C) stratified by detection method. WBC = white blood cell, APTT = activated partial thromboplastin time, PT = prothrombin time, INR = international normalised ration, ALT = alanine transaminase, AST = aspartate aminotransferase, CRP = C-reactive protein. Analysis by Kruskal Wallis test.*

Investigation	Total N		PCR positive	Serology positive only	p
Haemoglobin (g/L)	48	Median (IQR)	112.5 (105.5 to 121.0)	109.5 (101.8 to 113.2)	0.129
Haematocrit (%)	15	Median (IQR)	35.0 (31.9 to 37.6)	29.0 (29.0 to 30.6)	0.083
WBC (x10^9/L)	48	Median (IQR)	10.6 (7.2 to 15.6)	11.0 (8.8 to 15.7)	0.439
Lymphocyte count (x10^9/L)	48	Median (IQR)	0.9 (0.7 to 1.2)	1.2 (0.7 to 2.1)	0.368
Neutrophil count (x10^9/L)	48	Median (IQR)	8.3 (5.4 to 12.7)	8.6 (6.0 to 12.6)	0.544
Platelet count (x10^9/L)	48	Median (IQR)	182.5 (134.5 to 264.5)	161.0 (132.8 to 214.0)	0.464
APTT (sec)	35	Median (IQR)	35.0 (30.7 to 39.0)	34.0 (28.8 to 37.3)	0.529
PT (sec)	28	Median (IQR)	14.8 (13.4 to 15.8)	11.6 (11.0 to 12.0)	0.013
INR	11	Median (IQR)	1.1 (1.1 to 1.2)	1.2 (1.1 to 1.2)	0.772
ALT (IU/L)	43	Median (IQR)	27.0 (19.2 to 38.5)	45.0 (35.0 to 78.0)	0.010
Bilirubin (µmol/L)	43	Median (IQR)	10.0 (5.0 to 16.0)	8.0 (5.0 to 16.5)	0.980
AST (IU/L)	11	Median (IQR)	46.0 (36.5 to 79.5)	66.5 (51.0 to 83.2)	0.925
Glucose (mmol/L)	28	Median (IQR)	6.2 (5.2 to 6.4)	6.1 (5.7 to 7.5)	0.834
Urea (mmol/L)	43	Median (IQR)	4.2 (3.2 to 8.9)	4.5 (3.2 to 12.5)	0.518
Lactate (mmol/L)	24	Median (IQR)	1.3 (1.2 to 1.5)	1.2 (0.9 to 3.0)	0.663
Creatinine (µmol/L)	46	Median (IQR)	55.0 (36.0 to 73.0)	55.0 (38.0 to 102.0)	0.604
Sodium (mmol/L)	49	Median (IQR)	136.3 (133.0 to 138.0)	134.0 (132.0 to 135.0)	0.106
Potassium (mmol/L)	49	Median (IQR)	3.8 (3.5 to 4.2)	3.7 (3.2 to 3.9)	0.307
CRP (mg/L)	42	Median (IQR)	183.0 (135.0 to 277.0)	164.0 (81.0 to 252.0)	0.293
Platelet count < 150 (x 10^9/L)	48	No	19 (67.9)	13 (65.0)	1.000
		Yes	9 (32.1)	7 (35.0)	
Infiltrates on any CXR	38	No	4 (18.2)	10 (62.5)	0.008
		Yes	18 (81.8)	6 (37.5)	

Supplementary Figures

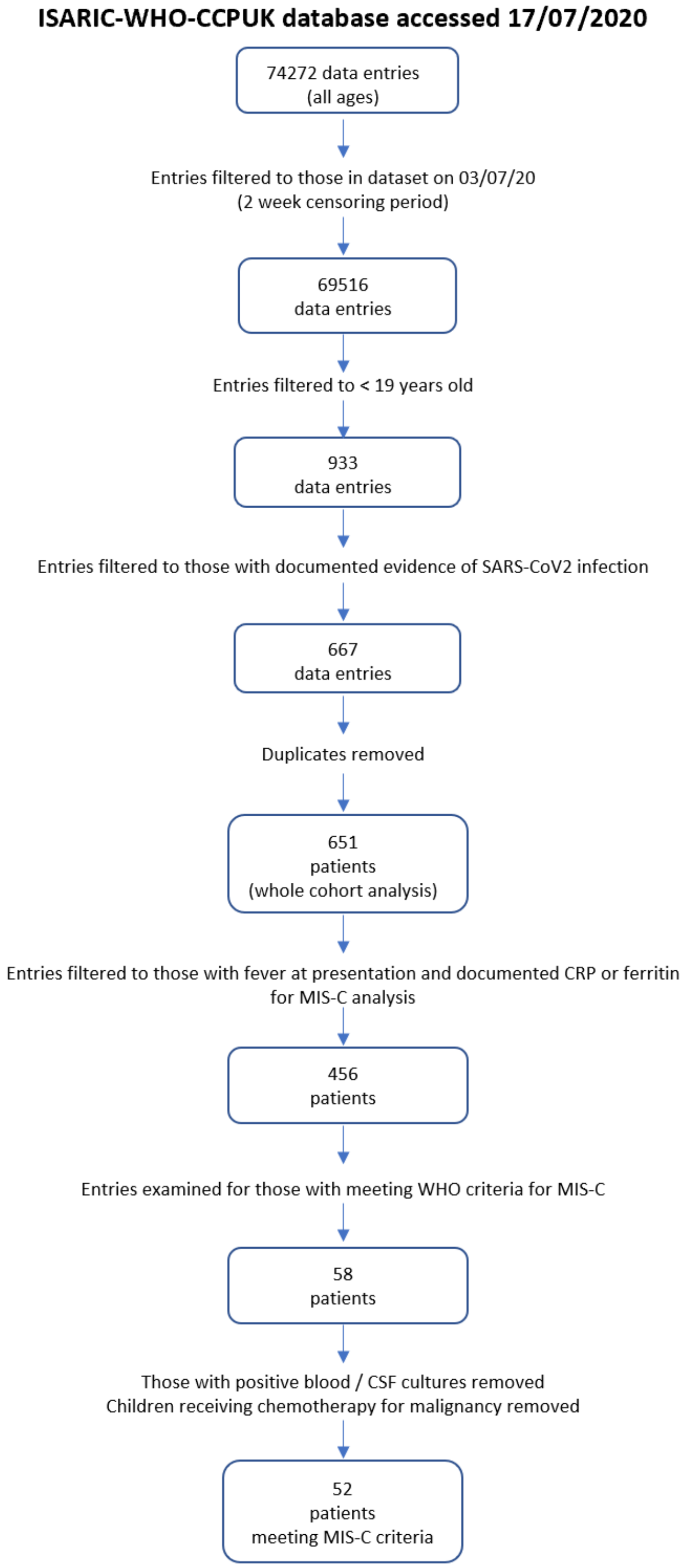

Supp Figure A. *Patient inclusion flowchart*

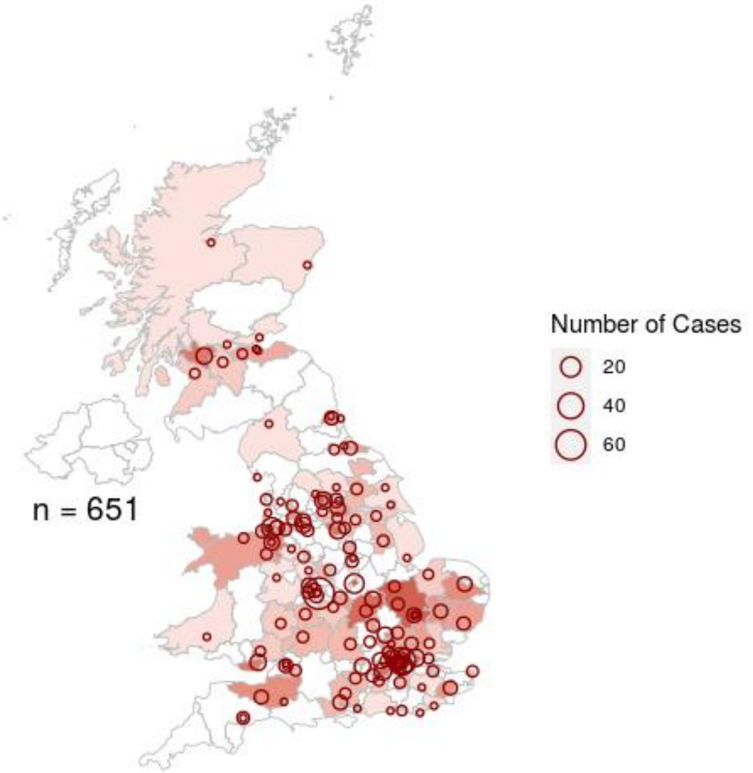

Supp Figure B. *Map of sites of patient enrolment and cases by site.*

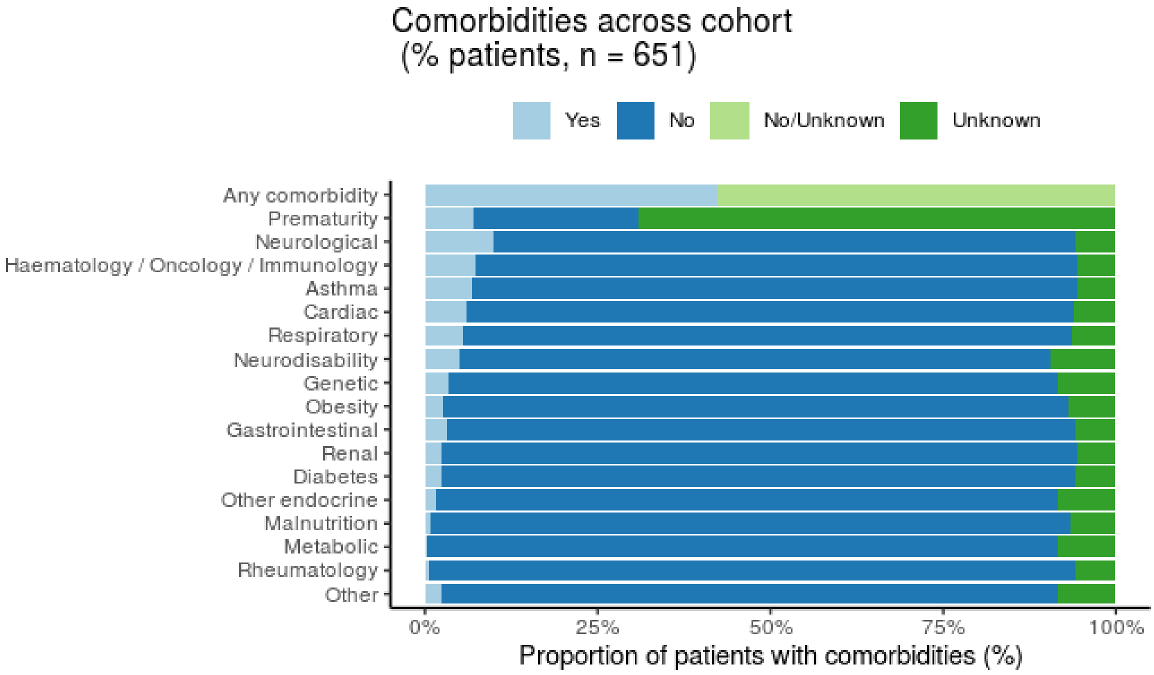

Supp Figure C. *Comorbidities across the whole cohort. Prematurity defined as gestation < 37 weeks. Prematurity data was only collected for children under 1 year old.*

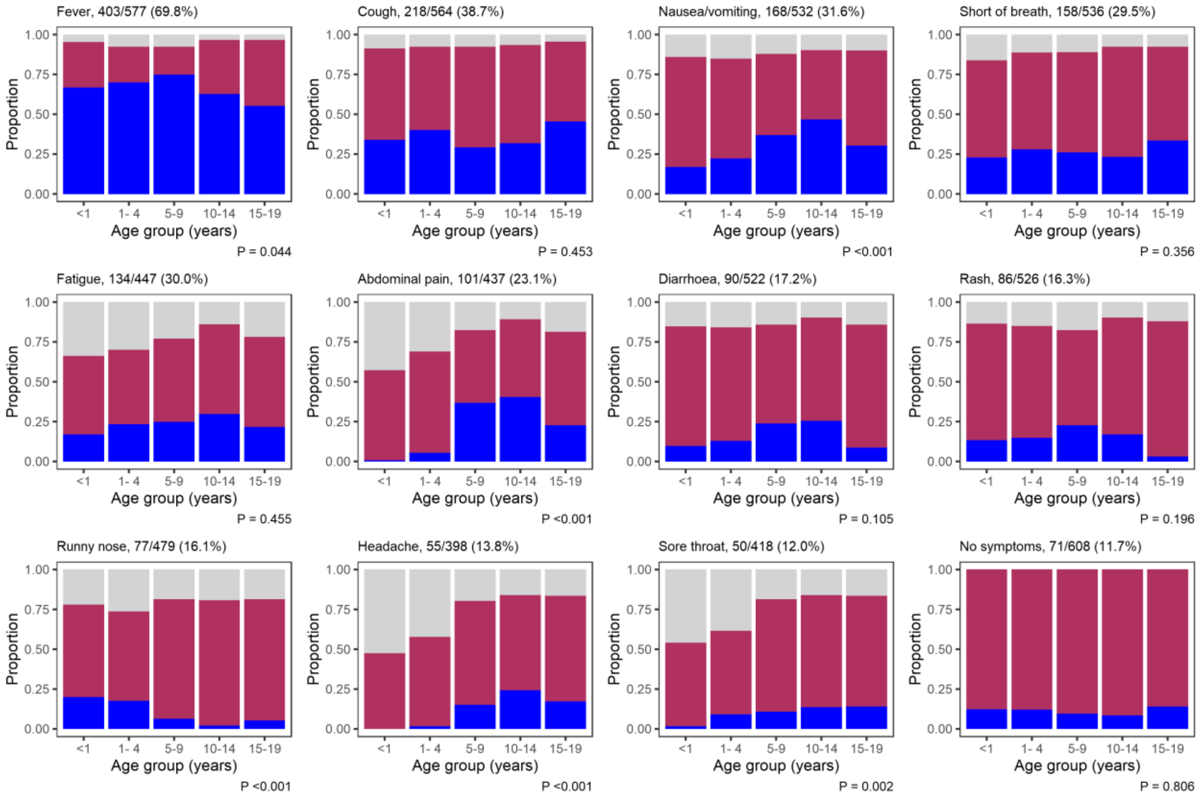

Supp Figure D. *Symptoms on admission to hospital by age. Blue bars represent patients with the symptom recorded present, maroon bars represent symptom recorded absent, and grey bars represent the symptom missing or unknown. Each plot is labelled with the symptom name, the number with the symptom present, the non-missing denominator, and the percentage excluding missing. P-values are from a chi-square test for trend.*

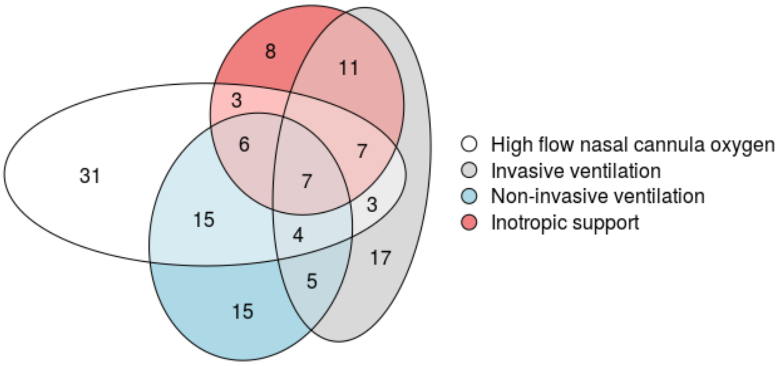

Supp Figure E. *Scale Euler diagram of treatments received across the whole cohort. (NB - Not all treatment combinations could be displayed in these intersections)*

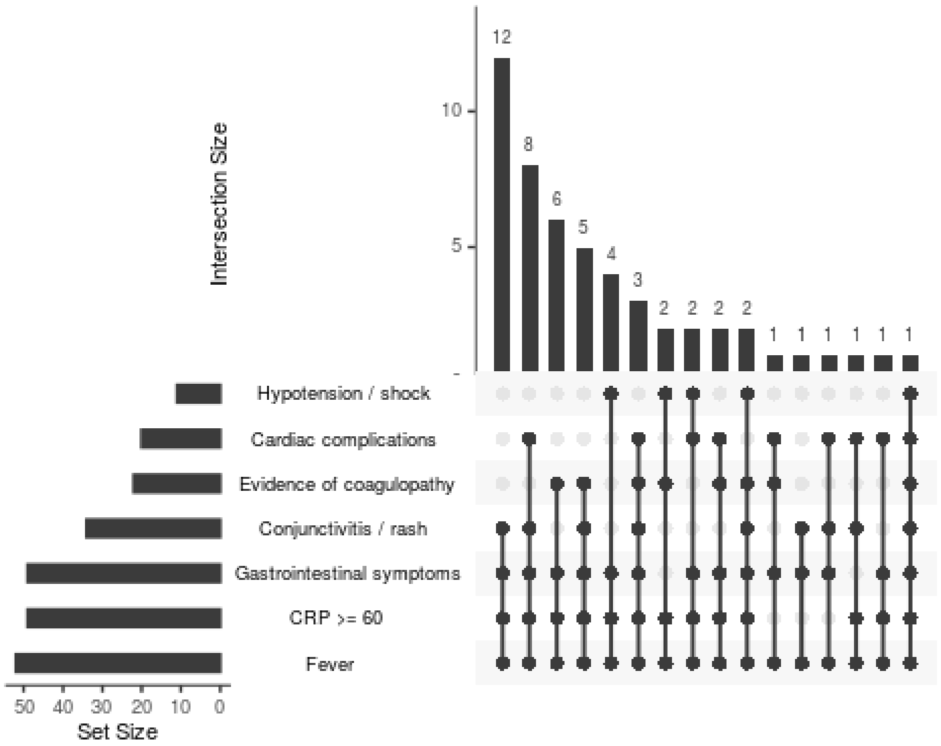

Supp Figure F. *UpSet plot of patients meeting Multisystem Inflammatory Syndrome in Children and Adolescents (MIS-C) WHO criteria. Plot represents a visualisation of set intersections in the data. CRP: C reactive protein.*

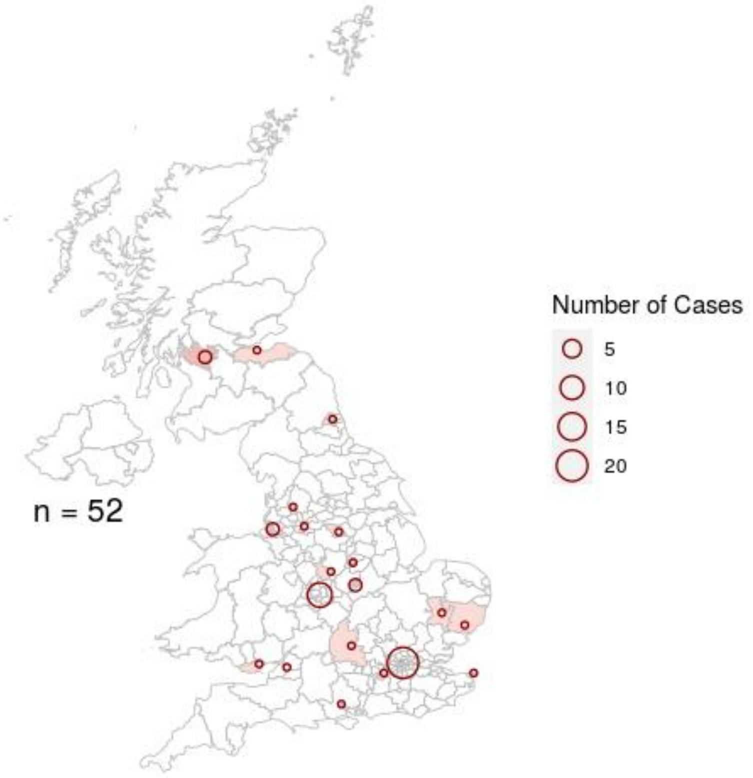

Supp Figure G. *Map of sites of origin for patients meeting criteria for Multisystem Inflammatory Syndrome in Children and Adolescents*

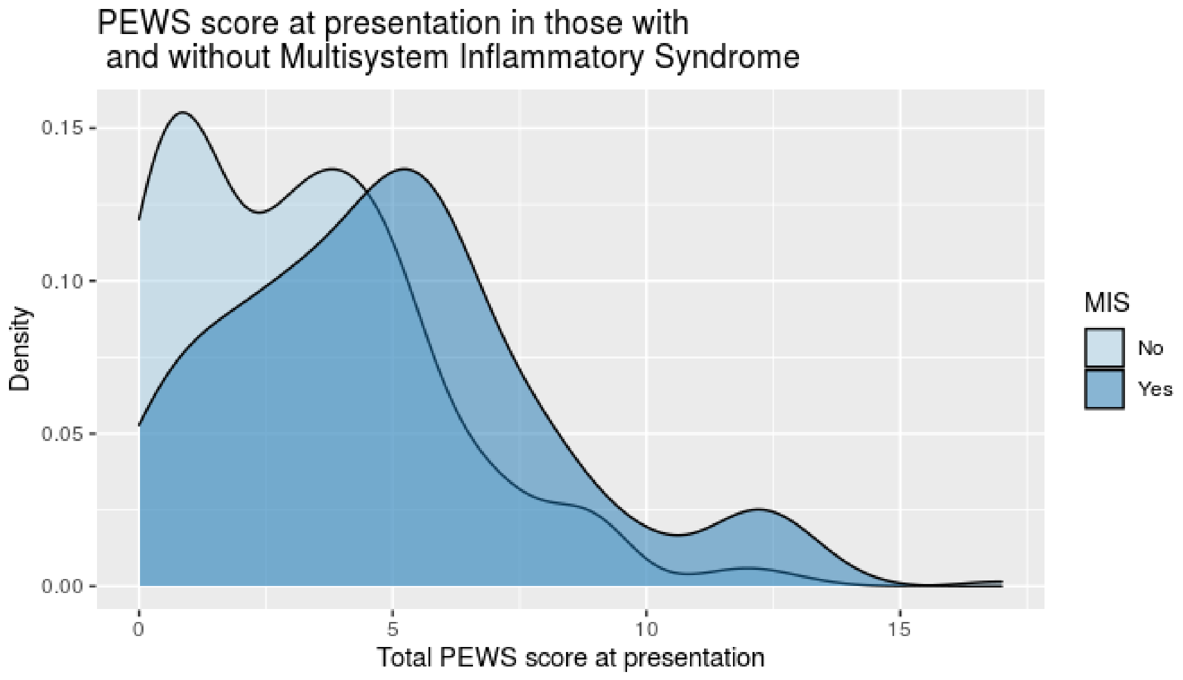

Supp Figure H. *Density plot of PEWS at presentation stratified by whether or not the patient met the criteria for Multisystem Inflammatory Syndrome. MIS-C = Multisystem Inflammatory Syndrome in Children and Adolescents.*

Supplementary References:

1. Ignjatovic V, Kenet G, Monagle P. Developmental hemostasis: recommendations for laboratories reporting pediatric samples. J Thromb Haemost [Internet]. 2012 [cited 2020 Aug 11];10(2):298–300. Available from: https://onlinelibrary.wiley.com/doi/abs/10.1111/j.1538-7836.2011.04584.x

2. Toulon P, Berruyer M, Brionne-François M, Grand F, Lasne D, Telion C, et al. Age dependency for coagulation parameters in paediatric populations. Results of a multicentre study aimed at defining the age-specific reference ranges. Thromb Haemost. 2016 04;116(1):9–16.

3. Arslan FD, Serdar M, Merve Ari E, Onur Oztan M, Hikmet Kozcu S, Tarhan H, et al. Determination of Age-Dependent Reference Ranges for Coagulation Tests Performed Using Destiny Plus. Iran J Pediatr [Internet]. 2016 Apr 30 [cited 2020 Aug 11];26(3). Available from: https://www.ncbi.nlm.nih.gov/pmc/articles/PMC4992153/

4. Williams MD, Chalmers EA, Gibson BES, Haemostasis and Thrombosis Task Force, British Committee for Standards in Haematology. The investigation and management of neonatal haemostasis and thrombosis. Br J Haematol. 2002 Nov;119(2):295–309.

5. Rokach L, Maimon O. Clustering Methods. In: Maimon O, Rokach L, editors. Data Mining and Knowledge Discovery Handbook [Internet]. Boston, MA: Springer US; 2005 [cited 2020 Aug 12]. p. 321–52. Available from: https://doi.org/10.1007/0-387-25465-X_15

6. Levandowsky M, Winter D. Distance between Sets. Nature [Internet]. 1971 Nov [cited 2020 Aug 12];234(5323):34–5. Available from: https://www.nature.com/articles/234034a0

7. Rousseeuw PJ. Silhouettes: A graphical aid to the interpretation and validation of cluster analysis. J Comput Appl Math [Internet]. 1987 Nov 1 [cited 2020 Aug 12];20:53–65. Available from: http://www.sciencedirect.com/science/article/pii/0377042787901257

8. Gates AJ, Ahn Y-Y. The Impact of Random Models on Clustering Similarity. J Mach Learn Res [Internet]. 2017 [cited 2020 Aug 12];18(87):1–28. Available from: http://jmlr.org/papers/v18/17-039.html

9. COVID-19: investigation and initial clinical management of possible cases [Internet]. GOV.UK. [cited 2020 Jul 7]. Available from: https://www.gov.uk/government/publications/wuhan-novel-coronavirus-initial-investigation-of-possible-cases/investigation-and-initial-clinical-management-of-possible-cases-of-wuhan-novel-coronavirus-wn-cov-infection
